# Supramolecular self-assembly in Traditional Chinese Medicine: molecular mechanisms, material basis of decoction efficacy, compatibility interpretation, and biomedical applications

**DOI:** 10.1186/s13020-026-01326-y

**Published:** 2026-01-13

**Authors:** Yinan Wang, Qiang Xiao, Can Liu, Wenjun He, Xinning Ren, Meifeng Xiao, Qijun He, Xue Pan, Fuyuan He

**Affiliations:** 1 School of Pharmacy, Hunan University of Chinese Medicine, Changsha, 410208 China; 2 Hunan Provincial Key Laboratory of Drugability and Preparation Modification of TCM, Changsha, 410208 China; 3 School of Integrated Chinese and Western Medicine, Hunan University of Chinese Medicine, Changsha, 410208 China

**Keywords:** Supramolecular self-assembly, Traditional Chinese Medicine, Pharmacological material basis, Compatibility interpretation, Efficacy enhancement and toxicity reduction, Biomedical applications

## Abstract

Traditional Chinese Medicine (TCM), characterized by multi-component and multi-target pharmacological effects, boasts millennia of clinical application. The complexity of its pharmacological material basis extends beyond individual chemical components to include physical structures formed among constituents. Supramolecular chemistry offers a new perspective for elucidating this basis. This review systematically summarizes recent advances in supramolecular self-assemblies within TCM, focusing on molecular formation mechanisms, key chemical component characteristics driving assembly, their roles in decoctions, and the supramolecular basis for "efficacy enhancement and toxicity reduction" in compatibility theory. The biomedical applications of supramolecular self‑assemblies in TCM are also reviewed. Ultimately, this work aims to offer new insights into TCM's material basis, clarify compatibility mechanisms, advance the supramolecular interpretation of TCM theories, and provide perspectives for TCM modernization and drug development.

**Graphical Abstract:**

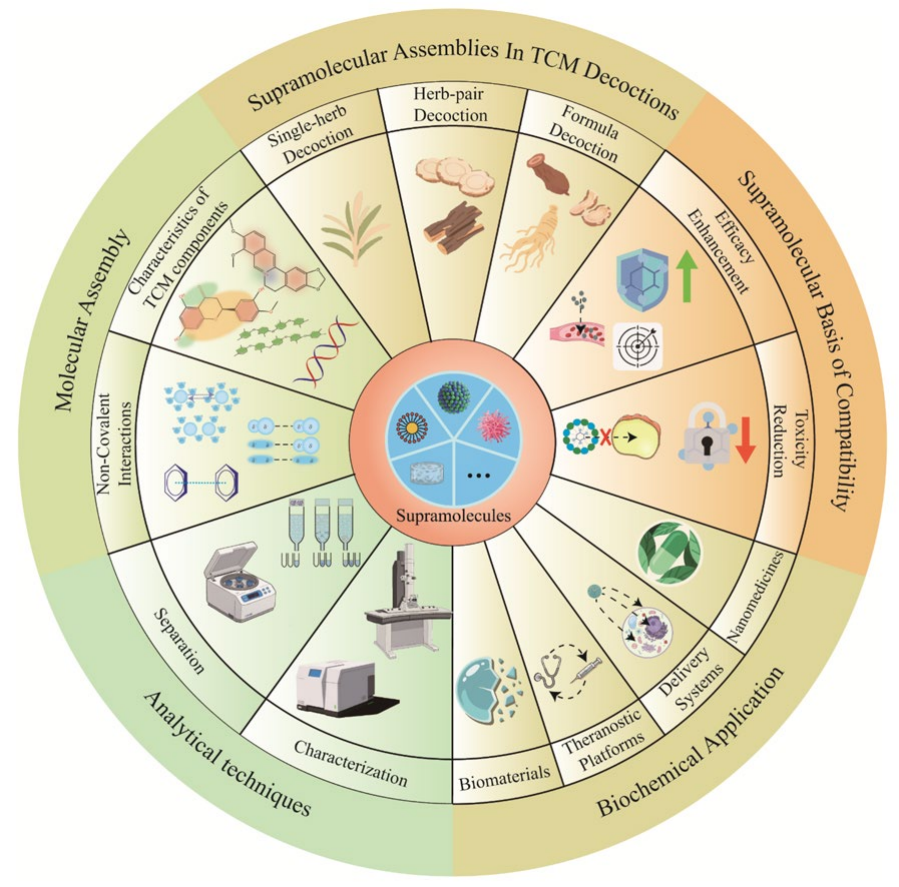

## Background

Traditional Chinese Medicine (TCM) boasts millennia of clinical application, with its efficacy verified through extensive practice [1]. Modern drugs developed from active components in TCM have significantly improved human health and quality of life. However, analysis solely at the chemical composition level is insufficient to fully elucidate TCM's complex material basis and mechanisms, as efficacy also depends on the physical states and structures formed through inter-component interactions. Supramolecules formed via non-covalent interactions in TCM have thus emerged as a crucial entry point for deciphering its material basis, with supramolecular chemistry providing a novel framework to understand the structural and functional distinctions of these assemblies from individual components.

Supramolecules refer to molecular assemblies formed by the self-assembly of two or more molecules through non-covalent interactions, which exhibit specific structures and functions [2]. This assembly process can significantly modulate key physicochemical properties of molecules, including solubility, reactivity, and biological activity [3, 4]. Supramolecular assemblies not only possess some characteristics of traditional polymers but also demonstrate unique behaviors such as self-healing, stimulus-responsiveness, and reversible polymerization [5–8]. Recent studies have shown that supramolecular assemblies are commonly found in TCM decoctions, and they exhibit clear pharmacological activity, expanding the understanding of the material basis of TCM efficacy [9]. Naturally derived TCM ingredients exhibit excellent biocompatibility and bioactivity; their structural complexity and diversity provide a robust foundation for supramolecular self-assembly. Compared to single ingredients, these assemblies show significant advantages in controlled and sustained release [9], targeted delivery [10], synergistic effects [11, 12], toxicity reduction [13], improved bioavailability [14], and stimulus-responsiveness [15].

With deepening research, the prevalence of supramolecular phenomena in TCM has been confirmed [16]. Recently, the self-assembly mechanisms of various bioactive ingredients in herbal medicine have been systematically reviewed [17]. Notably, He et al. pioneered the application of supramolecular chemistry to interpret TCM theory, proposing a potential supramolecular material basis for the meridian system, visceral organs, and the properties and efficacy of Chinese medicine, supported by preliminary experimental validation [18]. In recent years, researchers worldwide have conducted in-depth research on the composition, formation mechanisms, physical morphology, and pharmacological effects of supramolecules in TCM decoctions, providing new perspectives for elucidating TCM efficacy and theory [19–21]. Furthermore, research on designing self-assembled materials with diverse structures and functions based on supramolecular self-assembly principles using TCM active components is flourishing. This not only expands the scope of supramolecular research in TCM but also represents a crucial pathway toward practical applications.

This review aims to systematically summarize the research progress on TCM supramolecular self-assemblies, focusing on their molecular formation mechanisms, key characteristics of chemical components, existence forms, and pharmacological effects in TCM decoctions. It also explores the supramolecular basis of “efficacy enhancement and toxicity reduction” in compound compatibility and reviews their applications in biomedicine. Ultimately, this work seeks to offer novel insights into revealing the material basis and mechanisms of TCM efficacy, clarifying the scientific basis of compound compatibility, and promoting the development and translational application of supramolecular assembly-based TCM drugs.

## Molecular mechanisms of supramolecular self‑assembly in TCM

**Fig. 1:**
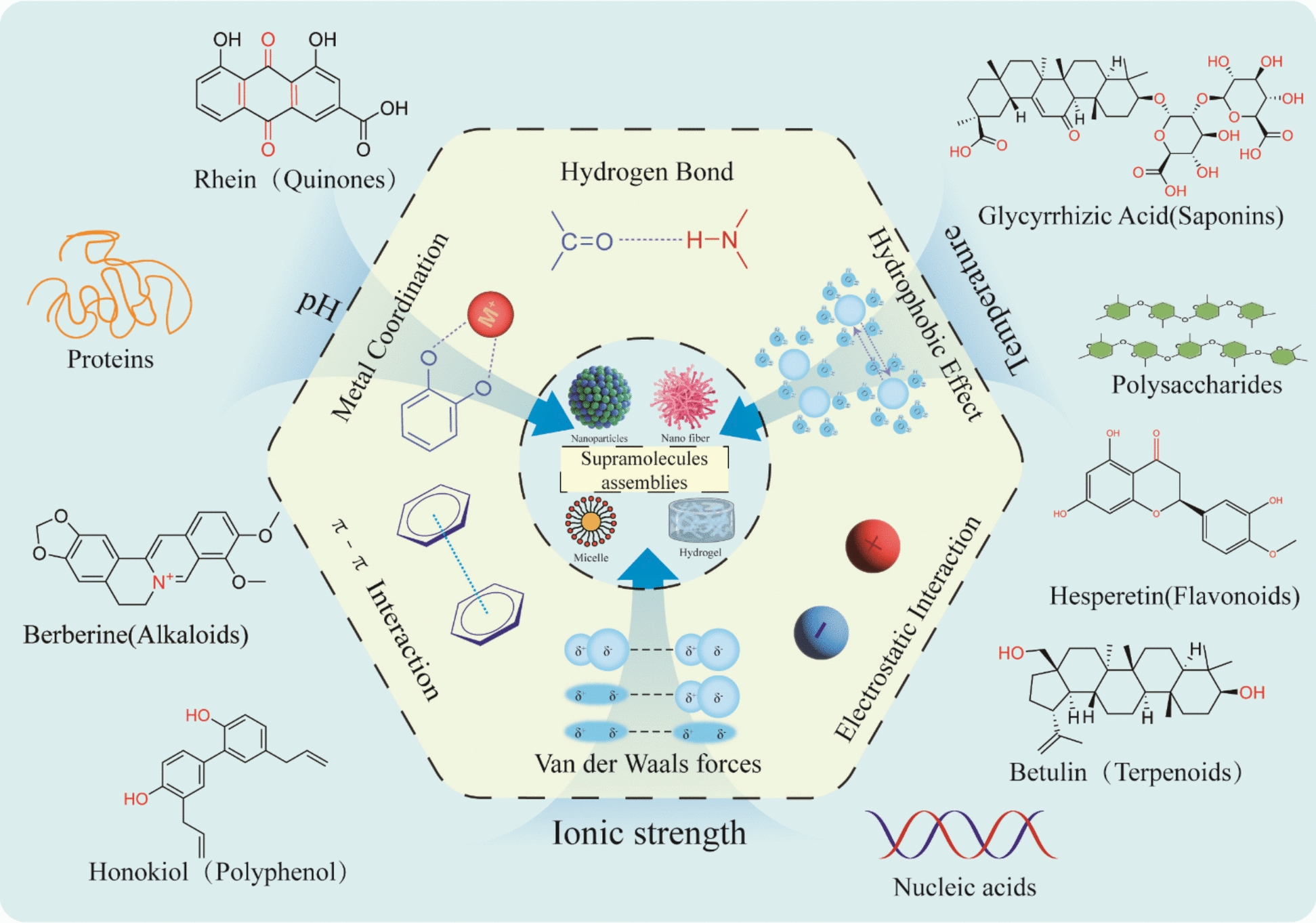
Schematic of supramolecular self-assembly in TCM. Diverse chemical components spontaneously organize through several non-covalent interactions—hydrogen bonding, hydrophobic effects, electrostatic forces, van der Waals interactions, π–π stacking, and metal coordination—to form supramolecular assemblies including nanoparticles, nanofibers, micelles, and hydrogels

Similar to other supramolecular compounds, supramolecular systems in TCM depend on diverse non‑covalent interactions to drive spontaneous molecular assembly and are modulated by external environmental factors such as pH, temperature, and ionic strength [22, 23]. TCM components comprise abundant primary metabolites as well as a wide diversity of secondary metabolites. These compounds typically contain distinct structural motifs and bioactive functional groups that, under appropriate conditions, spontaneously organize via these non-covalent forces into stable supramolecular architectures to exert their unique pharmacological effects (Fig. 1).

### Driving forces for supramolecular formation in TCM

Supramolecular assembly in TCM systems is driven by synergistic non-covalent interactions, primarily including hydrogen bonding, electrostatic interactions, hydrophobic effects, π-π stacking, van der Waals forces, and coordination bonding. Specific intermolecular interactions often dominate different categories of self-assembled structures. Notably, the reversible and dynamic nature of these forces not only regulates assembly behavior and structural stability but also profoundly dictates the physicochemical properties—such as solubility, membrane permeability, and stability—of the active ingredients. Therefore, a comprehensive understanding of the intrinsic characteristics of these interactions and their regulating factors, such as temperature, pH, and ionic strength, is essential. This knowledge elucidates the molecular basis and external conditions of supramolecular formation, revealing how microscopic interactions translate into macroscopic pharmacological effects.

Hydrogen bonding acts as a foundational driving force [24]. It arises from dipole–dipole interactions between a proton donor X–H and a proton acceptor Y. In TCM, components rich in hydroxyl or carboxyl groups (e.g., polyphenols, glycosides) utilize cooperative multi-hydrogen bonding to form topologically stable architectures [6]. Functionally, these networks can mask polar groups to enhance membrane permeability—for instance, the co-assembly of Baicalin (BA) and Berberine (BBR) transforms ionic monomers into amphiphilic nanoparticles, significantly improving transmembrane transport [25]. Similarly, exploiting the intermolecular hydrogen bonding typical of polyphenols, Epigallocatechin gallate (EGCG) co-assembles with Curcumin (Cur) to form core–shell nanoparticles. This interaction prevents premature metabolism and achieves intestine-targeted release, thereby increasing the oral bioavailability of Cur by 12-fold [26].

Electrostatic interactions manifest as attractive or repulsive forces between charged species. This force is the primary driver for the “efficacy enhancement and toxicity reduction” observed in many alkaloid-acid herb pairs. In TCM decoctions, pH variations dictate the ionization states of acidic and basic components. The formation of ion pairs or salt bridges neutralizes the charge of highly polar molecules, transforming them into amphiphilic complexes with enhanced lipophilicity and cellular uptake [27]. A compelling illustration is the electrostatic self-assembly of BA and BBR into nanoparticles (BA-BBR NPs), where the interaction between oppositely charged groups drives the formation of stable structures distinct from simple physical mixing [12]. This nanostructural modification generates a synergistic effect that significantly alleviates diarrhea-predominant irritable bowel syndrome by comprehensively regulating the microbiota-gut-brain axis, thereby achieving superior therapeutic outcomes compared to individual components.

The hydrophobic effect describes the entropy-driven aggregation of nonpolar moieties to minimize aqueous exposure. This is the critical mechanism by which TCM decoctions solubilize hydrophobic active ingredients. Specifically, amphiphilic components harness hydrophobic interactions to drive self-assembly into supramolecular assemblies, such as micelles or vesicles [28]. These structures create a nonpolar interior that encapsulates poorly water-soluble key active ingredients of TCM (such as Mangiferin or Cur) [20]. This “natural solubilization” mechanism explains why the aqueous solubility of hydrophobic components in compound decoctions is often tens to hundreds of times higher than in isolation.

*π-π* Stacking refers to attractive interactions between aromatic rings bearing π‑electron clouds [29, 30]. Since many TCM active ingredients possess aromatic structures, such as anthraquinones and flavonoids, this interaction is ubiquitous. Beyond structural stability, *π-π* stacking allows for high drug loading capacities and improves the physiological stability of assemblies [31]. For instance, during the co-assembly of Sanguinarine (SAN) and BA, strong *π-π* stacking occurs between the conjugated arene of Sanguinarine and the phenyl ring of Baicalin. This specific interaction drives the formation of a 'Z-type' layered offset arrangement, which acts as a critical structural adhesive to stabilize the cross-linked nanofiber superstructures [32]. Functionally, this binary supramolecular assembly exerts a synergistic antibacterial effect against MRSA by inhibiting bacterial virulence factors, while simultaneously accelerating wound healing through the modulation of local inflammatory responses.

Coordination bonding involves metal ions interacting with electron-donating groups. This interaction introduces stimuli-responsiveness to TCM assemblies [33]. Coordination bonds often exhibit pH sensitivity, allowing supramolecular nanomedicines to remain stable in the blood but dissociate in the acidic tumor microenvironment (TME), thereby achieving targeted drug release [34]. Additionally, metal–organic networks formed via coordination can scavenge Reactive Oxygen Species (ROS) [35], contributing directly to the antioxidant and anti-inflammatory efficacy of the decoction.

Van der Waals forces [36] encompass dipole–dipole, dipole-induced dipole, and London dispersion forces. Although individually weak, their cumulative effect is indispensable for the cohesive stability and structural compactness of TCM supramolecular assemblies [37]. These forces facilitate the aggregation of monomers into ordered structures, acting as a fundamental prerequisite for supramolecular formation. Consequently, van der Waals forces indirectly modulate the bioactivity of active ingredients by enabling the emergence of the supramolecular state, which exhibits distinct functional properties compared to free monomers.

Crucially, the formation of supramolecular assemblies depends not only on intrinsic molecular structures but also on external environmental parameters such as pH, temperature, and ionic strength [38–42] (Table 1). As detailed in Table 1, these environmental factors act as critical regulators of assembly stability and morphology. Specifically, pH can alter the protonation states of donors and acceptors, directly affecting the strength of hydrogen bonds and electrostatic attractions. Ionic strength modulates the electric double layer thickness, influencing the electrostatic repulsion between particles, while temperature variations can shift the thermodynamic equilibrium, favoring either hydrogen bonding (at lower temperatures) or hydrophobic interactions (at higher temperatures).

**Table 1:** Characteristics and influencing factors of non-covalent interactions driving TCM supramolecular assembly

Interaction	Key functional groups/structures	Binding energy range (kJ/mol)	Dominant molecular factors	Key environmental factors	Refs.
Hydrogen bonding	Proton donor X–H (O–H, N–H); Proton acceptor (C = O, N, F, π-systems)	10–40	Number and distribution of functional groups; Molecular flexibility and directionality	pH (protonation/deprotonation); Temperature; Solvent polarity	[6, 43]
Electrostatic interactions	Anions (COO⁻, O⁻, SO₃⁻); Cations (NH₃⁺, metal ions Fe^3^⁺, Ca^2^⁺, Mg^2^⁺)	5–350	Charge density and distribution; pKₐ/pKb values; Molecular geometry	pH; Ionic strength; Solvent dielectric constant	[44, 45]
Hydrophobic interaction	Alicyclic groups; aromatic rings; long alkyl chains	Entropy-driven, no fixed energy	Size of hydrophobic moieties; Amphiphilicity (hydrophilic-lipophilic balance); Surface properties	Temperature; Solvent polarity; Concentration	[46]
*π-π* stacking	Aromatic rings	1–50	Number of rings and extent of conjugation; Position of substituents	Solvent polarity; Temperature; Relative orientation of interacting moieties	[47]
van der Waals forces	Present in all molecules	< 5 (cumulative effects up to ~ 40)	Molecular polarizability and size; Strength of permanent/induced dipoles	Intermolecular proximity; Temperature; Pressure	[36, 48–50]
Coordination interaction	Metal ions (e.g., Fe^3^⁺, Zn^2^⁺); Lone pair-bearing groups (phenolic hydroxyl, carboxylate)	10–300	Nature and position of ligands; Coordination number; Steric configuration	pH; Ionic strength; Redox potential	[33]

In summary, the supramolecular assembly in TCM decoctions is centered on the synergy of multiple non-covalent driving forces, and this process is regulated by environmental factors. The combined effect of these two elements ultimately determines the assembly behavior of supramolecules and their corresponding pharmacological effects.

### Key features of representative components in TCM and their self-assembly mechanisms

**Fig. 2:**
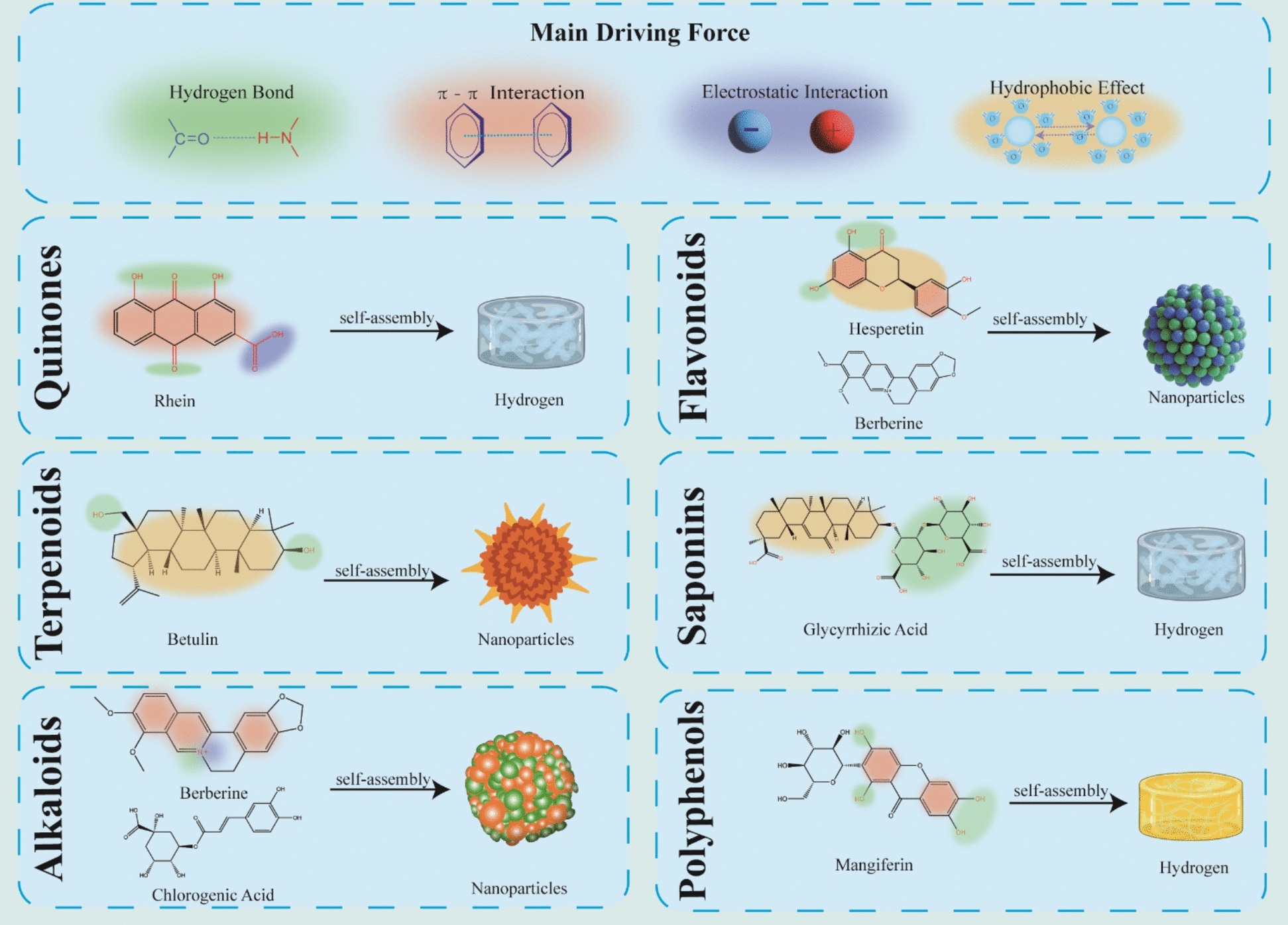
Self-assembly driving forces and representative nanostructures of small molecules derived from TCM

TCM comprises a vast array of structurally complex components, ranging from small molecules (Fig. 2) to macromolecules. These constituents are endowed with abundant functional groups that serve as the structural basis for intermolecular recognition. Driven by the non-covalent forces elucidated above, these diverse functional groups engage in specific cooperative interactions, thereby dictating distinct self-assembly modes and generating a wide variety of supramolecular architectures. Key features of representative components and their corresponding assembly mechanisms are summarized below.

#### Quinones

Quinones, characterized by an unsaturated cyclic diketone backbone, feature a core six-membered ring containing two conjugated double bonds and two carbonyl groups. Structurally, the pronounced polarity of carbonyl groups facilitates potent hydrogen bonding, while π-electron delocalization in the aromatic ring enables distinct *π-π* stacking. Additional carboxyl groups modulate intermolecular hydrogen-bond networks and electrostatic interactions, profoundly influencing self-assembly behavior. Collectively, these cooperative forces drive quinone self-assembly.

Rhein, a principal anthraquinone derived from *Rheum* species [51], contains two phenolic hydroxyl groups and one carboxyl group. Its anthraquinone backbone confers significant hydrophobicity. Investigations reveal that rhein’s self-assembly is driven primarily by *π-π* stacking, hydrogen bonding, and electrostatic interactions. Crucially, this process exhibits pH dependence: protonation/deprotonation states of carboxyl and hydroxyl groups dictate dominant noncovalent forces and final supramolecular architecture [9, 52]. Under acidic conditions (pH < 6.8), the molecule remains fully protonated, enhancing hydrophobicity through reduced carboxyl polarity and weakened electrostatic repulsion. Here, π-π stacking predominates, with hydrogen bonding assisting aggregation into spherical micelles, columns, or lamellar membranes, culminating in precipitation. As pH increases to the neutral-to-weakly alkaline range (pH 6.8–9.4), partial carboxyl deprotonation occurs while hydroxyl groups remain protonated, inducing amphiphilicity through the combination of a hydrophobic anthraquinone core and hydrophilic ionized moieties. In this regime, electrostatic attractions, hydrophobic forces, and hydrogen-bond networks collectively drive assembly. This progression follows a stepwise pathway: initiating with Na⁺-assisted dimer formation, followed by longitudinal π-π stacking into oligomers (trimers/tetramers), subsequent left-handed helical elongation into ~ 30 nm nanofibers, and finally cross-linking via hydrogen bonding and fiber–fiber electrostatics to yield a hydrogel. Under strongly alkaline conditions (pH > 9.4), complete deprotonation confers high negative charge density, enhancing hydrophilicity and electrostatic repulsion. Consequently, hydrophobic core-driven aggregation is disrupted, favoring formation of dispersed worm-like micelles stabilized by minimal hydrophobic interactions. The transition from nanofibers to hydrogels not only stabilizes the rhein molecules but potentially serves as a localized drug depot, extending the retention time of the active ingredient on mucosal surfaces and enhancing local bioavailability.

Moreover, the carbonyl and phenolic hydroxyl groups inherent to quinones serve as efficient coordination sites for metal ions, enabling construction of diverse metal-quinone supramolecular architectures [53].

#### Flavonoids and their glycosides

Flavonoids possess the 2-phenylchromone core structure, typically substituted with hydroxyl groups and frequently existing as glycosides. Their self-assembly is primarily governed by synergistic non-covalent forces arising from three key structural features: (i) molecular hydrogen bonding between hydroxyl and carbonyl groups, (ii) *π-π* stacking interactions mediated by aromatic rings, (iii) inherent amphiphilicity resulting from the hydrophobic flavonoid core conjugated with hydrophilic glycosyl moieties.

Hesperetin (HST), a flavanone predominantly found in *Citrus* species, self-assembles with BBR as reported by Gao et al. [54] initially, electrostatic attraction between the phenolic hydroxyl of HST and the quaternary ammonium cation of BBR forms an amphiphilic BBR-HST complex. In this complex, the hydrophobic flavanone and isoquinoline rings orient inward, while freely accessible HST hydroxyl groups extend outward, establishing the primary amphiphilic conformation. Progressive temperature or concentration changes drive *π-π* stacking between the aromatic rings of HST and BBR in a face-to-face orientation, while hydrogen bonding stabilizes alignment between the methoxy group of HST and the methylenedioxy group of BBR. Concurrently, hydrophobic interactions induce further core association and hydrophilic group exposure, collectively promoting transition towards a lower energy state. Following a lamellar intermediate phase, these amphiphilic units progressively aggregate and coil via hydrogen-bond networks and hydrophobic forces, ultimately self-assembling into spherical nanoparticles approximately 170–180 nm in diameter. Functionally, these self-assembled nanoparticles exhibit superior therapeutic efficacy against ulcerative colitis compared to their physical mixtures. This enhancement is attributed to the assembly's synergistic ability to regulate the immune microenvironment and repair the damaged intestinal barrier, specifically by upregulating tight junction proteins, including ZO-1 and occludin, thereby overcoming the critical limitations of poor aqueous solubility and low oral bioavailability inherent to the individual monomers.

BA, a flavonoid glycoside primarily isolated from *Scutellariae* Radix, undergoes self-assembly with BBR as described by Huang et al. [25] Electrostatic attraction between BA’s weakly ionized carboxylic acid group and BBR's quaternary ammonium cation generates the amphiphilic BBR-BA complex, featuring an inward-oriented hydrophobic core (BA’s flavonoid moiety and BBR’s isoquinoline ring) and outward-directed hydrophilic glucuronosyl group. During gradual cooling, hydrophobic interactions and *π-π* stacking direct molecular reorganization, while enhanced carboxylic acid ionization further strengthens electrostatic forces, facilitating transition to a lower-energy state. Subsequently, the amphiphilic complexes further aggregate through hydrogen bonding to form nanoparticles ranging from 100 to 250 nm. Functionally, this transformation from ionic monomers into amphiphilic nanoparticles significantly improves the membrane permeability of BA. As noted in Sect. 2.1, such co-assembled nanodrugs demonstrate superior efficacy in regulating the gut microbiota compared to physical mixtures.

#### Terpenoids

Terpenoids constitute a class of compounds built from isoprene units and are classified into monoterpenes, sesquiterpenes, or triterpenes based on their oligomerization degree [55]. Triterpenoids exhibit particularly pronounced self-assembly behavior. Their assembly mechanism originates from synergistic interactions between hydrophobic domains and hydrophilic moieties within their molecular architecture. Hydrophobic backbones and planar conformations facilitate hydrophobic interactions, while hydrophilic groups establish intermolecular hydrogen-bonding networks.

Betulin—a pentacyclic dihydroxy triterpenoid isolated from *Betula papyrifera* bark—self-assembles via terminal hydroxyl groups forming robust intermolecular hydrogen bonds [56]. Concurrently, its rigid pentacyclic scaffold provides additional driving forces through hydrophobic interactions and van der Waals forces. Under hydrogen-bond dominance, molecules adopt head-to-tail arrangements that organize into lamellar assemblies. These lamellae subsequently curl into nanofibers, which laterally aggregate forming petal-like sheets radially arranged around fibrous cores. Ultimately, this hierarchical assembly yields three-dimensional flower-like architectures.

Ursolic acid (UA)—a pentacyclic triterpenoid abundant in *Eriobotryae japonica* and *Ligustrum lucidum* foliage—initially forms ~ 150 nm spherical nanoparticles driven by hydrophobic interactions [57]. Terminal hydroxyl/carboxyl groups enhance nanoparticle stability through hydrogen bonding. Partial carboxyl deprotonation generates carboxylates, creating a surface potential of ~ −7 mV through balanced electrostatic repulsion and hydrophobic attraction that prevents excessive aggregation and ensures excellent colloidal stability. Consequently, nanoparticles remain stably dispersed via synergistic hydrophobic forces and hydrogen bonds. Functionally, these carrier-free nanoparticles exhibit potent antitumor activity and liver-protective effects. By blocking the COX-2/VEGFR2/VEGFA pathway and activating CD4 + T cells, they significantly inhibit tumor growth while modulating the immune microenvironment.

Wang et al. [58] reported that oleanolic acid and glycyrrhetinic acid (GRA) self-assembly involves hydroxyl/carboxyl groups constructing hydrogen-bond networks while their hydrophobic triterpenoid skeletons undergo parallel stacking. This generates reverse-oriented dimers that further stack through skeletal hydrophobic interactions. Subsequent molecular aggregation ultimately yields monodisperse nanospheres ~ 300 nm in diameter. Functionally, this co-assembly elicits a synergistic antitumor effect by combining the distinct cell cycle arrest mechanisms of its components. Additionally, it serves as a hepatoprotective agent by upregulating antioxidant levels, thereby reducing systemic toxicity.

#### Saponins

Saponins consist of triterpenoid or steroid aglycones covalently bonded to oligosaccharide chains through glycosidic linkages. Their propensity for self-assembly originates principally from their amphiphilic structure: the hydrophobic aglycone core mediates molecular aggregation via hydrophobic interactions, while the hydrophilic sugar chains facilitate hydration shell formation and participate in hydrogen bonding. Moreover, ubiquitous chiral centers within the triterpenoid aglycone further modulate molecular packing, and its planar conformation may enhance assembly stability through *π-π* stacking [59]. A representative triterpenoid saponin is glycyrrhizic acid (GA), isolated from *Glycyrrhizae* Radix. Saha et al. [60] elucidated the self-assembly mechanism of GA in aqueous environments. Functioning as an amphiphilic natural saponin, GA initiates self-assembly driven by its hydrophobic triterpenoid aglycone (18β-GRA) and hydrophilic disaccharide glucuronic acid. Hydrophobic interactions predominantly govern aglycone stacking, whereas the hydrophilic groups engage water molecules through a hydrogen-bonding network. GA self-assembly proceeds concentration-dependently in a stepwise manner: Upon exceeding 0.05 wt%, monomers associate, forming dimers/oligomers that ultimately elongate into uniform, ultralong fibrils. Leveraging the inherent chirality of its aglycone, these fibrils adopt a right-handed helical morphology across the entire concentration range (0.05–2 wt%). At concentrations reaching 0.1 wt%, fibrils undergo parallel alignment, constituting a nematic liquid crystal phase. At concentrations ≥ 0.3 wt%, fibril cross-linking induces a three-dimensional network, yielding a transparent hydrogel. Functionally, this hierarchical self-assembly fundamentally transforms the physicochemical identity of GA, converting discrete monomers into a mechanically stable, viscoelastic network. This structural evolution transcends the capabilities of the single-molecule form by generating a macroscopic material with distinct rheological properties and long-range order. Consequently, the formation of such supramolecular architectures provides a strategy to enhance the inherent utility of natural saponins, offering advantages in bioavailability and biocompatibility that expand their therapeutic potential beyond simple monomeric administration.

#### Alkaloids

Alkaloids constitute a predominant class of nitrogen-containing natural products extensively distributed in plant systems. Attributable to their characteristic structural elements—notably the basic nitrogen atoms and aromatic rings—alkaloids demonstrate multi-level self-assembly capabilities: protonated nitrogen atoms mediate electrostatic attraction with anionic groups, establish hydrogen-bonding networks with proton donors, and engage aromatic rings in *π-π* stacking, thereby enabling stable intermolecular associations.

Among the most representative alkaloids exhibiting self-assembly behavior is BBR, the principal active component of *Coptidis* Rhizoma. Its inherent benzylisoquinoline scaffold and quaternary ammonium group naturally furnish the required multimodal driving forces. Fu et al. [61] elucidated the self-assembly of BBR with chlorogenic acid (CGA) in aqueous media, forming nanoparticles. The underlying mechanism encompasses: (i) electronic interactions between the aromatic core of BBR and the phenyl ring of CGA; (ii) hydrogen bonding between BBR's tertiary nitrogen and CGA's hydroxyl/carboxyl groups; and (iii) electrostatic attraction between BBR's quaternary ammonium cation and the carboxylate anion of CGA. Similarly, Huang et al. [62] described BBR/cinnamic acid (CA) self-assembly, predominantly governed by: (i) hydrogen bonding between BBR's nitrogen and CA's carboxyl group, generating a Butterfly-like Unit Cell; (ii) *π-π* stacking of their respective aromatic rings, yielding a lamellar 3D architecture. As summarized in Table 2, natural compounds co-assembling with BBR predominantly feature negatively charged functionalities, which electrostatically complement BBR's positively charged groups. These electrostatic co-assemblies often mask the intense bitterness of native alkaloids and shield the quaternary ammonium group from premature metabolic clearance, ultimately improving patient compliance and oral bioavailability.

**Table 2:** Natural compounds co-assembling with berberine (BBR) and their self-assembly driving forces

Alkaloid	Molecular structure	Co-assembling compound	Molecular structure	Primary driving forces	Refs.
Berberine		Baicalin		Electrostatic interactions; *π-π* stacking; Hydrophobic interactions	[25]
		Wogonoside		Electrostatic interactions; Hydrophobic interactions	[63]
		Rhein		*π-π* stacking, Electrostatic interactions; Hydrogen bonding	[11]
		Tannic acid		Electrostatic interactions; *π-π* stacking; Hydrogen bonding	[64]
		Paclitaxel		*π-π* stacking; Hydrophobic interactions	[65]
		Cinnamic acid		*π-π* stacking; Hydrogen bonding	[62]
		Chlorogenic acid		Electrostatic interactions; *π-π* stacking; Hydrogen bonding	[61]
		Aristolochic acid		Electrostatic interactions; *π-π* stacking; Hydrophobic interactions	[66]

Moreover, SAN—a benzophenanthridine quaternary ammonium alkaloid extracted from Macleaya cordata—possesses functional groups analogous to BBR and exhibits distinctive self-assembly characteristics. Wang et al. [32] characterized the SAN/BA co-assembly pathway. Initially, electrostatic interaction between the carboxylate of BA and the quaternary ammonium cation of SAN forms amphiphilic V-type ion-pair units. Subsequent *π-π* stacking between the phenyl ring of BA and the conjugated arene of SAN produces Z-type assemblies adopting a layered, offset stacking arrangement. Ultimately, hydrogen bonding between the hydroxyls of BA and the methylenedioxy groups of SAN further stabilizes a fiber network, culminating in a 3D cross-linked nanofiber superstructure. Functionally, this carrier-free hydrogel exerts a potent synergistic antibacterial effect against MRSA, surpassing the efficacy of individual components. It significantly promotes wound healing by inhibiting bacterial virulence factors and alleviating the inflammatory response, demonstrating distinct advantages over traditional antibiotic therapies.

#### Polyphenols

The core structure of polyphenols comprises multiple phenolic hydroxyl groups attached to aromatic rings. Phenolic hydroxyls elicit intermolecular networks via hydrogen bonding, whereas the rigid benzene rings afford structural stabilization through *π-π* stacking. These synergistic interactions underpin polyphenol self-assembly. Functionally, the resulting assembly serves as a protective barrier for the labile phenolic hydroxyls, preventing oxidative damage and preserving their bioactivity.

Mangiferin (MF), a C-glucoside xanthone primarily isolated from *Mangifera indica* and *Anemarrhena asphodeloides*, demonstrates self-assembly driven by hydrogen bonding and *π-π* stacking [67]. Specifically, hydrogen bonds between MF's hydroxyl groups and the glycosidic oxygen propel directional alignment along a single axis. Concurrently, the xanthone ring achieves stabilization through anti-parallel alignment, forming H-type assemblies. MF oligomers, stabilized by these cooperative forces, serve as fundamental building blocks for nanofiber assembly, culminating in a three-dimensional network. From a therapeutic perspective, this self-assembled state accelerates diabetic wound healing by promoting the transition of macrophages from the pro-inflammatory M1 phenotype to the anti-inflammatory M2 phenotype and enhancing angiogenesis.

Honokiol (HK), a biphenolic compound sourced from *Magnolia* bark, self-assembles into uniform spherical nanoparticles [68]. Its assembly evolves through three synergistic forces: (i) Hydrogen bonding between phenolic hydroxyls drives anisotropic (1D) assembly; (ii) hydrophobic interactions within the biphenyl core promote 3D aggregation; (iii) aromatic ring *π-π* stacking contributes supplementary stabilization. Molecular dynamics (MD) simulations indicate that this assembly pathway minimizes the total system energy; hydrogen bonding predominates as the primary driver, with hydrophobic and *π-π* stacking serving auxiliary structural roles. Functionally, these nanoparticles act as a potent immunotherapeutic agent for P53-mutated colorectal cancer. They effectively reprogram tumor-associated macrophages (TAMs) from the M2 to the M1 phenotype and activate T cells, thereby suppressing tumor growth and metastasis.

Gallic acid, a ubiquitous polyphenolic acid in sources like tea leaves and pomegranates, similarly undergoes cooperative self-assembly with Resveratrol [69]. Resveratrol's dual aromatic rings synergize with gallic acid, driving the emergence of fibrous supramolecular hydrogels exhibiting substantial mechanical strength. Hydrogen bonding constitutes the predominant directing force in fiber formation, while *π-π* stacking provides crucial secondary stabilization. Therapeutically, this co-assembled system exhibits significant antibacterial activity against S. aureus and E. coli. By combining the antioxidant and anti-inflammatory properties of its components, it creates a favorable environment that promotes the healing of bacterially infected wounds.

Notably, polyphenols containing ortho-dihydroxy structures can also undergo self-assembly via metal coordination. For instance, tannic acid coordinates with Fe(III) to construct a stable network at the interface. This coordination-driven assembly effectively shields therapeutic cargos from gastric degradation, thereby significantly enhancing their oral bioavailability [70].

#### Metal ions

Trace metal ions in TCM, such as Fe^2^⁺/^3^⁺, Zn^2^⁺, Cu^2^⁺, and Ca^2^⁺, can coordinate with specific functional groups in bioactive TCM components or synergize with other noncovalent interactions. These interactions participate in or drive the supramolecular assembly process, fundamentally modulating the structure and function of the assemblies.

For instance, in the Baihu Decoction (BHD), metal ions (Ca^2^⁺, Mg^2^⁺, Zn^2^⁺) spontaneously assemble with GA and MF via electrostatic interactions, hydrogen bonding, and hydrophobic effects, forming spherical nanoparticle assemblies (~ 100 nm diameter). These natural supramolecular carriers substantially enhance the aqueous solubility of MF and provide effective delivery for poorly soluble compounds [20]. Similarly, Pi et al. [21] demonstrated that in the Ma Xing Shi Gan Decoction (MXSGT), Ca^2^⁺ interacts with ephedrine, GRA, and amygdalin to form spherical supramolecular nanoparticles, further establishing the essential role of metal ions in TCM aggregate formation.

Beyond naturally occurring assemblies, implementing metal coordination strategies for constructing natural product-based nanomedicines has yielded highly efficacious systems. Fe^3^⁺, 5-aminolevulinic acid, and Cur self-assemble through coordination bonds, *π-π* stacking, and hydrogen bonding, yielding pH-responsive full-active pharmaceutical ingredient (full-API) nanodrug. This nanomedicine facilitates targeted drug release within acidic tumor microenvironments while simultaneously enhancing systemic stability and therapeutic efficacy under physiological conditions [34]. Li et al. [71] employed Zn^2^⁺ coordination coupled with noncovalent interactions to mediate the assembly of Cur with 9-fluorenylmethoxycarbonyl-L-histidine, constructing controllable nanoparticles with enhanced biological stability and tumor targeting. Furthermore, Feng et al. [53] developed a Shikonin-based Fe^3^⁺-polyphenol metal-phenol network framework. This approach not only confers uniform spherical nanoparticles and robust colloidal stability but also demonstrates potent capabilities for multifunctional drug delivery.

In summary, within both the inherent supramolecular assemblies of classic TCM decoctions and engineered metal-natural product nanomedicine systems, trace metal ion coordination serves as a pivotal driver of supramolecular self-assembly and structural/functional optimization.

#### Biomacromolecules

Beyond the small-molecule components of TCM, large-molecule natural products—such as proteins, polysaccharides, and nucleic acids—also display distinctive self-assembly behaviors. Their complex spatial configurations and diverse functional group arrangements endow these biomacromolecules with the capacity to assemble functional nanostructures through the concerted modulation of multiple noncovalent interactions.

Proteins primarily utilize their amino acid residue functional groups—including nonpolar side chains, hydroxyl, amide, carbonyl, imino, alongside charged moieties (-COO⁻, -NH₃⁺, guanidino, imidazole)—to generate the hydrophobic, hydrogen bonding, electrostatic, and other noncovalent interactions driving self-assembly. For instance, *Pseudostellariae* Radix protein assembles into sub-100-nm spherical nanoparticles upon heat and pH modulation. Its assembly mechanism entails heat-induced partial unfolding of *Pseudostellariae* Radix protein monomers, which exposes hydrophobic groups and mediates initial aggregation via hydrophobic interactions—mimicking the protein denaturation dynamics occurring during TCM decoction. Subsequently, pH adjustment from pH 7.0 to 5.50–5.90 fine-tunes intermolecular attractive/repulsive forces through electrostatic interactions, preventing excessive aggregation while promoting structural reorganization of intermediate oligomers into uniformly dispersed assemblies. At pH 5.70, synergistic hydrogen bonding and hydrophobic interactions enable assembly into spherical nanoparticles, dramatically augmenting the aqueous solubility and stability of encapsulated Cur [72]. Similarly, *Armeniacae* Semen Amarum protein undergoes heat-induced self-assembly into nanoparticles, primarily driven by subunit rearrangement and specific noncovalent forces. Following 95 °C heat denaturation, disruption of the heterodimeric 11S globulin subunit cleaves interchain disulfide bonds, releasing acidic and basic polypeptides. Acidic polypeptides then reassemble via disulfide bonds into homodimers, which further organize into spherical nanoparticles through hydrophobic interactions and hydrogen bonding. Crucially, electrostatic balance stabilizes assembly, yielding monodisperse nanoparticles [73]. Collectively, these studies demonstrate that protein conformational rearrangement induced by stimuli like heat or pH exposes hydrophobic domains, thereby facilitating nanostructure assembly.

Moreover, natural proteins can construct highly symmetric supramolecular architectures—including nanowires, nanorings, and superlattices—via metal ion coordination or host–guest recognition, exhibiting multifunctional potential for light harvesting, enzymatic catalysis, and drug delivery [74].

Polysaccharides leverage their profuse hydroxyl groups to establish extensive hydrogen-bonding networks. Their chain conformation also permits self-assembly driven by hydrophobic association and spatial constraints. *Angelicae sinensis* Radix polysaccharides (APS), for instance, co-assemble with GA forming liver-targeting nanocarriers [75]. This assembly originates from APS-provided hydrogen bonding and hydrophobic interactions, while surface-exposed saccharide units enable tumor cell recognition, enhancing delivery specificity. Following the cooling phase subsequent to high-temperature decoction, the *Coptidis* Rhizoma decoction undergoes spontaneous self-assembly. Natural polysaccharides present within the decoction, predominantly *Coptidis* Rhizoma polysaccharide, aggregate into nanoparticles ranging from 100 to 500 nm in diameter, exhibiting surface charges between −7 and −18 mV. Crucially, these self-assembled nanoparticles remarkably enhance the intestinal absorption of BBR. In aqueous ethanol, *Codonopsis* Radix polysaccharides undergo competitive hydration, which compresses their hydration layer and exposes hydrophobic regions, inducing random aggregation. Concurrently, hydroxyl and carboxyl groups consolidate intermolecular binding via hydrogen bonding. Furthermore, entangling high-molecular-weight *Codonopsis* Radix polysaccharides chains reinforce the 3D network, generating a physically crosslinked gel with a porous architecture. This system accommodates both hydrophilic and hydrophobic drugs, demonstrating shear-thinning, self-healing, injectability, and sustained co-release capabilities [76].

Nucleic acids serve as anionic polyelectrolytes possessing an inherently rigid duplex structure. They form supramolecular complexes with cationic lipids, polyamines, etc., via electrostatic interactions, adopting mesophases including lamellar or hexagonal arrangements [77], whose conformation and function respond to environmental cues such as pH and hydration [78].

#### Exosome-like nanovesicles

**Fig. 3:**
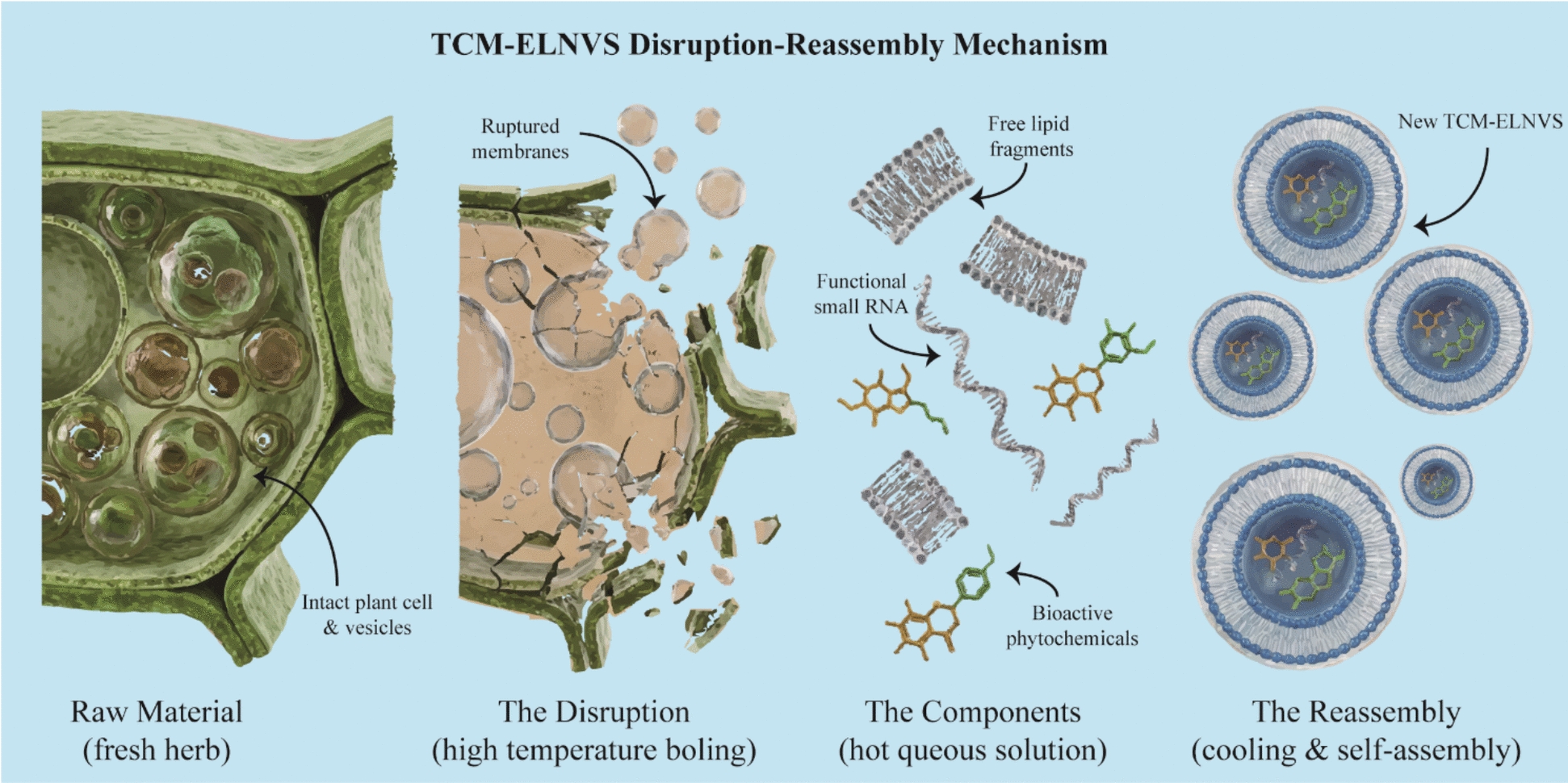
The "Disruption-Reassembly" mechanism of TCM-derived Exosome-like Nanovesicles (ELNVs) formed during high-temperature decoction

Unlike native plant-derived extracellular vesicles (PDEVs), whose isolation technologies, composition, and biological functions have been extensively summarized [79], exosome-like nanovesicles (ELNVs) found in TCM decoctions represent a distinct class of supramolecular assemblies generated through high-temperature processing. While native PDEVs function as physiological transporters [80, 81], TCM-derived ELNVs are characterized by their "disruption-reassembly" formation mechanism (Fig. 3b). Research indicates that during boiling, endogenous vesicles in raw herbs initially undergo disruption, followed by spontaneous supramolecular reassembly to form novel vesicular structures. These post-boiling vesicles differ fundamentally from the native vesicles inherently present in plant tissues.

From a supramolecular perspective, these ELNVs are thermodynamically stable systems selected or formed under specific decoction conditions. For instance, Song et al. [82] investigated the Yiqi Huoxue Jiedu Decoction (YQHXJDD), a complex TCM formula containing dried herbs like Astragalus membranaceus and Curcuma zedoaria. They demonstrated that an optimized decoction protocol (40 min high-heat followed by 30 min low-heat) maximized the yield of self-assembled ELNVs. These reassembled nanovesicles exhibited high colloidal stability (zeta potential of -35.52 mV) and encapsulated specific bioactive cargo, including lipids (34.98% of metabolites) and functional small RNAs.

Functionally, these supramolecular assemblies serve as potent therapeutic carriers. The YQHXJDD-derived ELNVs significantly inhibited ovarian cancer growth and metastasis in vivo. Mechanistically, they remodeled the immune microenvironment by increasing TNF-α levels, promoting the polarization of M1 macrophages, and elevating the CD4 + /CD8 + T cell ratio. These findings confirm that ELNVs in TCM are not merely biological residues but are functional, heat-stable supramolecular nanomedicines generated through the interaction of lipids, RNAs, and other phytochemicals during the decoction process.

### Separation and characterization strategies for TCM supramolecular assemblies

Elucidating the molecular mechanisms of supramolecular self-assembly, as described in previous sections, necessitates the efficient isolation and precise characterization of these assemblies from the complex matrices of TCM decoctions. Unlike the extraction of free small molecules, the isolation of supramolecular assemblies requires the preservation of their non-covalent structural integrity.

Separation techniques commonly applied in TCM research include differential centrifugation and size-exclusion chromatography (SEC), which effectively fractionate nano-assemblies based on size and molecular weight [83], distinguishing them from free monomers and macroscopic precipitates [19, 84]. Dialysis is frequently employed as a subsequent purification step to remove interfering small molecules while retaining the intact assembly structures [20, 85].

Characterization strategies are essential for validating structure–function relationships and identifying the driving forces of assembly. Morphological assessment routinely relies on scanning electron microscopy (SEM) and transmission electron microscopy (TEM) to visualize the size and topological features of these nanostructures [16, 19]. Notably, cryo-electron microscopy (cryo-EM) has emerged as a pivotal technique. A groundbreaking study by Peng et al. successfully resolved the molecular structure of self-assembled nanofibrils formed by the saponin Zingibroside R1 at an atomic resolution of 2.5 Å using cryo-EM [86]. This work, for the first time, revealed the precise spatial arrangement and specific non-covalent interactions (such as hydrogen bonding) that define the architecture of a natural TCM supramolecular assembly, marking a critical transition in the field from simple morphological observation to precise structural analysis.

Furthermore, confirming the specific non-covalent interactions (discussed in Sect. 2.1) requires a multimodal analytical approach. Spectroscopic methods, including Fourier-transform infrared (FTIR) spectroscopy, ultraviolet (UV) spectroscopy, and nuclear magnetic resonance (NMR) spectroscopy, are indispensable for identifying functional groups involved in interactions such as hydrogen-bonding networks and π-π stacking [87]. Thermodynamic techniques like isothermal titration calorimetry (ITC) provide quantitative data on binding energetics [88–91], while MD simulations offer atomistic insights into dynamic assembly pathways and interaction mechanisms [92]. This integrated methodology—encompassing isolation, morphological imaging, and interaction validation—collectively provides the experimental foundation for confirming supramolecular structures in TCM.

## Supramolecular structures in TCM decoctions and their pharmacological research

TCM decoctions represent a historically prioritized dosage form, in which boiling maximizes the dissolution of bioactive constituents and enhances oral bioavailability—thereby enabling broad clinical utility for complex diseases [93]. Critically, beyond water-soluble chemicals, these decoctions contain self-assembled micro/nanoscale supramolecular assemblies formed during aqueous extraction; these assemblies exhibit defined pharmacological activities [19, 94]. Research on these supramolecular assemblies transcends the pharmacological limitations of single-component analysis, thereby illuminating the holistic synergy and material basis of TCM efficacy. This section systematically examines supramolecular self-assembly and their therapeutic roles in single-herb, herb-pair, and multi-herb formula decoctions.

### Supramolecular research on single-herb decoctions

Single-herb medicines, serving as the fundamental units of TCM compound formulas, harbor structurally diverse chemical components. During decoction, elevated temperatures induce the self-assembly of these components into supramolecular assemblies via characteristic functional groups, a phenomenon universally observed in single-herb decoctions. Zhuang et al. [16] employed dynamic light scattering (DLS) and TEM to confirm the presence of such assemblies ranging from nanometers to micrometers in size across 60 single-herb decoctions. Recent investigations have focused on elucidating the specific forms, composition, and pharmacological activities of these supramolecular assemblies within select single-herb decoctions (Table 3).

**Table 3:** Supramolecular characteristics and bioactive functions in single-herb decoctions

Single-herb	Decoction functions	Supramolecular assemblies (Morphology, Size, Zeta Potential)	Supramolecular key constituents	Bioactive functions	Refs.
*Isatidis* radix	Antiviral; Anti-inflammatory; clearing heat; detoxify	Spherical nanoparticles; particle size: 57–300 nm	Polysaccharides; proteins	Promote normal cell proliferation; inhibit cancer cell/macrophage growth; enhance anti-H1N1 activity	[84, 97]
*Coptidis* Rhizoma	Clearing heat/dampness; detoxify	Irregular nanoparticles; particle size: 264.8–427.4 nm; zeta potential: − 6.85 to − 18.2 mV	Polysaccharides; proteins	Promote intestinal absorption and bioavailability of BBR; modulate tight junctions; activate endocytosis pathways	[98]
*Puerariae lobatae* Radix	Improve microcirculation; cardiovascular protection	Spherical nanoparticles; particle size: 200–351 nm	Puerarin; daidzein; daidzin; genistein; polysaccharides; proteins	Enhance therapeutic efficacy; promote component absorption; enable synergistic action	[99]
*Glycyrrhiza* Radix et Rhizoma	Mediating compatibility; anti-inflammatory	SPHERICAL nanoparticles; particle size: 74.1 nm; zeta potential: 24.3 mV	Proteins	Function as drug carriers; promote cell proliferation; enhance drug solubility	[100]
*Lonicerae japonicae* Flos	Clearing heat; detoxify; anti-inflammatory	Spherical nanoparticles; particle size: 47.6–273.8 nm; zeta potential: − 11.7 mV	Esterified pectin; phenolic acids; flavonoids; proteins	Enhance antioxidant activity; improve stability; promote functional synergy	[101]
*Pseudostellariae* Radix	Tonify *Qi*; enhance immunity	Spherical nanoparticles; particle size: 131.4 nm	Proteins	Stimulate splenocyte proliferation; promote immune factor secretion	[102, 103]
*Rabdosiae rubescentis* Herba	Antithrombotic; anti-inflammatory	Spherical nanoparticles; particle size: 4.5–234.3 nm	Nucleotides; phenolic acids; diterpenoids	Enhance drug delivery; inhibit sP-selectin release; exert antithrombotic effect	[104]

The *Isatidis* Radix (Ban-Lan-Gen) decoction (BLGD) is broadly employed for treating infectious and inflammatory diseases, including influenza, acute hepatitis, herpes, and viral encephalitis [95]. *Isatidis* Radix contains abundant amphiphilic molecules such as proteins and polysaccharides. Upon boiling, these amphiphilic species are released and self-assemble into nanoparticles through non-covalent interactions. Researchers isolated six nanoparticle fractions (F1-F6) with sizes ranging from 57 to 300 nm from BLGD. These polysaccharide-dominated nanoparticles exhibited robust aqueous stability. Critically, the F2-F5 nanoparticles demonstrated significant antiviral efficacy against the H1N1 influenza virus, with activity at 0.4 mg/mL surpassing that of the drug ribavirin. These results establish BLGD nanoparticles as a crucial contributor to its antiviral activity [84].

The *Coptidis* Rhizoma decoction, traditionally used to manage damp-heat syndromes, bacterial dysentery, and digestive inflammation, forms nanoassemblies primarily composed of polysaccharides and proteins during boiling and cooling [96]. These structures are irregularly shaped, measuring 264.8–427.4 nm in size and exhibiting negative surface charge. Notably, these assemblies substantially enhance the intestinal absorption of the poorly soluble bioactive compound BBR by modulating the intestinal barrier and promoting cellular uptake. Mechanistically: (i) The negatively charged assemblies modulate tight junction integrity in intestinal epithelial cells, increasing paracellular permeability to BBR. (ii) They facilitate BBR transcellular transport via GA energy-dependent active transport, clathrin-mediated endocytosis, and macropinocytosis. This provides compelling scientific evidence supporting the superior clinical efficacy of TCM decoctions compared to isolated bioactive compounds.

Furthermore, diverse supramolecular architectures with distinct pharmacological functions have been characterized in other single-herb decoctions. For instance, in the decoction of *Lonicerae japonicae* Flos, a herb critical for clearing heat and detoxification, spherical nanoparticles ranging from 47.6 to 273.8 nm were identified. Compositional analysis revealed that these assemblies are driven by the interaction of esterified pectin with phenolic acids and flavonoids. Functionally, these supramolecular structures not only improve the stability of the active ingredients but also exhibit enhanced antioxidant activity compared to the monomeric components, suggesting a functional synergy arising from assembly. Similarly, the *Pseudostellariae* Radix decoction, used for tonifying *Qi*, contains spherical protein-based nanoparticles. These assemblies have been shown to stimulate splenocyte proliferation and promote the secretion of immune factors, thereby providing a material basis for the herb's immunomodulatory effects.

Table 3 summarizes the compositions, morphological features, and bioactive functions of these and other single-herb assemblies. As detailed in the table, these assemblies exhibit bioactive functions—such as enhanced drug delivery, immune regulation, and synergistic therapeutic effects—that correlate directly or indirectly with the efficacy of their corresponding decoctions. This correspondence suggests that supramolecular assemblies constitute integral functional components contributing to the bioactivity of single-herb formulations.

### Supramolecular research in TCM herb-pair decoctions

Herb pairs, representing core dyadic combinations systematically developed through TCM theory and clinical practice, exemplify specific therapeutic synergism, typically achieved via “mutual assistance” (*xiang xu*) or “mutual enhancement” (*xiang shi*) principles. These pairs constitute ideal model systems for investigating TCM compatibility mechanisms and form the fundamental prototype for compound formulations. Co-decoction of paired herbs facilitates interactions between phytochemicals of distinct botanical origins. Compared to homologous compound self-assembly in single-herb decoctions, this process significantly expands the dimensionality and complexity of molecular interactions, establishing a critical foundation for studying multi-herb compatibility.

*Glycyrrhizae* Radix et Rhizoma, one of the most prevalent TCM herbs, routinely forms herb pairs. Supramolecular phenomena in such paired decoctions are pervasive. For instance, the *Glycyrrhizae* Radix et Rhizoma-*Coptidis* Rhizoma pair elicits heat-clearing and detoxifying effects. Li et al. [91] demonstrated that acidic *Glycyrrhizae* Radix et Rhizoma components and alkaline *Coptidis* Rhizoma components co-assemble into spherical supramolecular nanoparticles during decoction. GA and BBR were identified as key structural motifs driving assembly, significantly potentiating BBR’s inhibitory efficacy against *Staphylococcus aureus* and establishing these nanoparticles as the material basis for antibacterial activity. Characterization revealed these nanoparticles exhibited substantially enhanced antibacterial efficacy versus mechanical mixtures or non-supramolecular fractions, achieving a bacteriostatic rate of 55.75% at 500 μg/mL with distinct membrane disruption; biofilm eradication reached 72.09% at 750 μg/mL. Analogous supramolecular assemblies with critical bioactivities are likewise observed in other Glycyrrhizae Radix et Rhizoma-containing herb pairs. The *Glycyrrhizae* Radix et Rhizoma-*Zingiberis* Rhizoma pair—employed for lung atrophy, asthma, and pollakiuria—forms supramolecular assemblies via GA and 6-gingerol co-assembly [105], exhibiting augmented antioxidant activity and enhanced absorption compared to monomers. Similarly, supramolecular assemblies in *Glycyrrhizae* Radix et Rhizoma-*Aconiti* Lateralis Radix Praeparata and *Glycyrrhizae* Radix et Rhizoma-*Paeoniae* Radix Alba decoctions mitigate inherent toxicity or enhance component bioavailability. Details of their supramolecular characteristics and bioactive functions are summarized in Table 4.

**Table 4:** Supramolecular characteristics and bioactive functions in TCM herb-pair decoctions

Herb pair	Decoction functions	Supramolecular assemblies (Morphology, Size, Zeta Potential)	Supramolecular key constituents	Bioactive functions	Refs.
*Glycyrrhizae* Radix et Rhizoma-*Coptidis* Rhizoma	Clearing heat; detoxify	Spherical nanoparticles; particle size: 161.6 nm	Flavonoids; alkaloids; triterpenoids	Antibacterial activity; disrupts bacterial biofilms	[91]
*Glycyrrhizae* Radix et Rhizoma-*Zingiberis* Rhizoma	Warms spleen/lung	Irregular assemblies; particle size: 162.3 nm; zeta potential: −8.94 mV	6-Gingerol; GA	Increases 6-Gingerol solubility; increases antioxidant capacity; promotes in vitro release	[105]
*Glycyrrhizae* Radix et Rhizoma-*Aconiti* Lateralis Radix Praeparata	Warms *yang*; disperses cold; relieves pain	Colloidal particles; particle size: 238.20 nm	Glycoproteins; aconitine derivatives	Reduces aconitine toxicity	[112]
*Glycyrrhizae* Radix et Rhizoma-*Paeoniae* Radix Alba	Antispasmodic; analgesic	Irregular spherical nanoparticles; particle size: 223.16 nm	GA; liquiritin; albiflorin; paeoniflorin; benzoylpaeoniflorin	Increases ileal absorption of albiflorin, paeoniflorin; benzoylpaeoniflorin	[113]
*Coptidis* Rhizoma-*Scutellariae* Radix	Clearing heat; detoxify; antibacterial; anti-inflammatory	Spherical nanoparticles; particle size: 100–250 nm; zeta potential: −22.2 mV	BBR; BA	Increases antibacterial activity; disrupts bacterial membranes; inhibits biofilm; modulates amino acid metabolism	[25]
*Coptidis* Rhizoma-*Polygalae* Radix	Clearing heat/dampness; detoxify; tranquilizes	Spherical nanoparticles; particle size: 92.6 nm; zeta potential: −31.6 mV	BBR; 3,4,5-trimethoxycinnamic acid	Potent antibacterial activity against MRSA	[106]
*Coptidis* Rhizoma-*Mume* Fructus	Anti-inflammatory	Spherical nanoparticles; particle size: 98.3 nm; zeta potential: −16.3 mV	BBR; CGA	Increases anti-inflammatory effect; inhibits pyroptosis; modulates NF-κB and caspase-11 pathways	[107]
*Scutellariae* Radix*-Hedyotidis* Herba	Anti-cancer	Spherical nanoparticles; particle size: 130–200 nm	Aurantiamide acetate; scutebarbatine A; glyceryl palmitate	Increases anti-tumor activity; increases drug targeting; decreases systemic toxicity	[108]
*Astragali* Radix-*Angelicae sinensis* Radix	Tonifies *Qi* and blood	Irregular spherical nanoparticles; particle size: 133–279 nm; zeta potential: −21 mV	Saponins; flavonoids; phthalides; organic acids	Inhibits myocardial fibrosis; improves endothelial function; sustains release of actives	[109]
*Rhodiolae crenulatae* Radix et Rhizoma-*Taraxaci* Herba	Anti-fatigue; antioxidant; clearing heat; detoxify	Nano-vesicles	Small molecules; peptides; sRNAs; sphingolipids	Antifibrotic/anti-inflammatory; oral nucleic acid delivery	[111]
*Anemarrhenae* Rhizoma-*Phellodendri chinensis* Cortex	Treats type 2 diabetes	Spherical nanoparticles; particle size: 225.9 nm; zeta potential: −13.00 mV	Small-molecules; polysaccharides	Increases drug stability; sustains drug release; increases oral bioavailability of anti-diabetic actives	[110]

*Coptidis* Rhizoma, a cornerstone herb for heat-clearing, damp-drying, fire-purging, and detoxification, is commonly integrated to achieve “efficacy enhancement and toxicity reduction” or broaden therapeutic indications. For example, the combination of *Coptidis* Rhizoma and *Scutellariae* Radix—both possessing heat-clearing/damp-drying properties—synergistically intensifies these effects. Huang et al. [25] documented that BBR (from *Coptidis* Rhizoma) and BA (from *Scutellariae* Radix) self-assemble into nanoparticles during the traditional co-decoction process, forming a homogeneous stable gel. Functional assessment indicated that these NPs notably increased antibacterial efficacy against *S. aureus* by disrupting membrane integrity, inhibiting biofilm formation, and impairing amino acid metabolism. Additionally, BBR and 3,4,5-trimethoxycinnamic acid in the *Coptidis* Rhizoma-*Polygalae* Radix pair co-assemble into antibacterial assemblies [106], while BBR and CGA in *Coptidis* Rhizoma-*Mume* Fructus form supramolecular assemblies that enhance anti-inflammatory activity [107].

The *Scutellariae* Radix*-Hedyotidis* Herba pair (SH) is extensively utilized in oncology therapeutics, with a long-standing history in empirical clinical formulations. Zhu et al. [108] isolated spherical nanoparticles (SH-NPs) through co-decoction in 70% ethanol–water. Compositional analysis identified scutebarbatine A (from *Scutellariae* Radix), aurantiamide acetate (from *Hedyotidis* Herba), and glyceryl palmitate (from *Hedyotidis* Herba) as primary components. SH-NPs potently inhibited MDA-MB-231 proliferation, surpassing unpurified SH extract efficacy. In murine xenograft models, high-dose SH-NPs significantly suppressed tumor growth without observable toxicity. Further mechanistic studies demonstrated that SH-NPs enhance the tumor targeting of active components primarily through the enhanced permeability and retention (EPR) effect, thereby exerting potent antitumor activity.

Beyond these interactions involved in toxicity reduction or solubility enhancement, supramolecular assembly in herb pairs also plays a pivotal role in optimizing pharmacokinetics and therapeutic targeting. In the *Astragali* Radix-*Angelicae sinensis* Radix pair, a classic combination for tonifying *Qi* and blood, saponins, flavonoids, and phthalides co-assemble into irregular spherical nanoparticles. These assemblies enable the sustained release of active ingredients and have been shown to attenuate myocardial fibrosis by inhibiting the endothelial-to-mesenchymal transition (EndMT) pathway [109]; similarly, the *Anemarrhenae* Rhizoma-P*hellodendri chinensis* Cortex pair forms spherical nanoparticles stabilized by polysaccharides. These supramolecular structures significantly enhance the stability of the active constituents and increase the oral bioavailability of therapeutics for type 2 diabetes [110]. Additionally, the *Rhodiolae crenulatae* Radix et Rhizoma-*Taraxaci* Herba pair yields novel sRNA-loaded nanovesicles that function as natural nucleic acid carriers, conferring anti-fibrotic and anti-inflammatory effects [111].

Critically, experimental evidence demonstrates that the supramolecular assemblies formed during the decoction of herb pairs are closely associated with their traditional therapeutic effects, as detailed in Table 4.

### Supramolecular research in TCM formula decoctions

TCM formula decoctions constitute complex heterogeneous systems guided by the sovereign-minister-assistant-messenger (Jun-Chen-Zuo-Shi) principles, integrating three or more medicinal herbs. Unlike homologous self-assembly in single-herb decoctions or limited interaction networks, multi-herb formulas exhibit substantially greater constituent diversity and interaction complexity. The resulting supramolecular assemblies demonstrate multi-component synergy while enabling dynamically modifiable biological functions. Elucidating the formation mechanisms, structural attributes, and inherent relationship between these assemblies and the holistic pharmacological efficacy is thus foundational to understanding TCM compatibility doctrine and compound formulation mechanisms. Recent advances in characterizing supramolecular assemblies within canonical TCM decoctions are exemplified by systematic investigations of the following representative formulas (Table 5):

**Table 5:** Supramolecular characteristics and bioactive functions in TCM formula decoctions

Formula name	Formula composition	Decoction functions	Supramolecular assemblies (Morphology, Size, Zeta Potential)	Supramolecular key constituents	Bioactive functions	Refs.
Huanglian Jiedu decoction	*Coptidis* Rhizoma; *Scutellariae* Radix; *Phellodendri chinensis* Cortex; *Gardeniae* Fructus	Clearing heat and toxins; anti-inflammatory; neuroprotective	Spherical nanoparticles; particle size: 174 nm	BA; BBR	Neuroprotective effect; against CoCl₂-induced toxicity in PC12 cells	[94, 114]
Baihu decoction	*Anemarrhenae* Rhizoma; *Glycyrrhizae* Radix et Rhizoma;*Oryzae* Semen, Gypsum	Clearing heat; antipyretic	Spherical nanoparticles; particle size: 100 nm; zeta potential: −3.11 mV	Neomangiferin; MF; GA; ammonium glycyrrhizinate; Inorganic ions (Ca^2^⁺, Mg^2^⁺, Zn^2^⁺); saponins; polysaccharide; starch	Enhanced antipyretic effect; increased solubility of poorly soluble actives; reduced inflammatory cytokine levels	[20, 115]
Maxing Shigan decoction	*Ephedra* Herba; *Glycyrrhizae* Radix et Rhizoma; *Armeniacae* Semen Amarum;Gypsum	Clearing lung heat; anti-inflammatory; diuretic	Spherical nanoparticles; particle size: 50–150 nm	Ephedrine; pseudoephedrine	Antipyretic; anti-inflammatory; enrichment of actives; synergistic efficacy; modulates cell proliferation	[19, 21, 117]
Mahuang Fuzi decoction	*Ephedrae* Herba; Glycyrrhizae Radix et Rhizoma;*Aconiti* Lateralis Radix Praeparata	Anti-inflammatory; antipyretic; analgesic	Spherical nanoparticles; particle size: 123.5 nm	Alkaloids; flavonoids; terpenoids	Inhibits inflammatory mediators and NF-κB pathway; modulates arginine metabolism	[119]
Xiebai San decoction	*Mori* Cortex; *Lycii* Cortex;*Glycyrrhizae* Radix et Rhizoma; *Oryzae* Semen	Clearing lung heat	Spherical nanoparticles; particle size: 104.53 nm; zeta Potential: −5.14 mV	Kukoamine B; mulberroside A; liquiritin	Increased oral bioavailability of actives; delayed clearance; enhanced efficacy	[85]
Naoluo Xintong decoction	*Astragali* Radix; *Chuanxiong* Rhizoma; *Notoginseng* Radix et Rhizoma; *Gastrodiae* Rhizoma;*Carthami* Flos; *Angelicae sinensis* Radix; *Scolopendra*	Promoting blood circulation; neuroprotective	Irregular nanoparticles; particle size: 185.5–353.13 nm; zeta potential: −16.07 to −20.57 mV	Polysaccharides; proteins; saponins	Enhanced resistance to oxidative stress; inhibition of apoptotic pathways; regulation of neuronal function	[121]
Ge-Gen-Qin-Lian decoction	*Puerariae Lobatae* Radix; *Scutellariae* Radix; *Coptidis* Rhizoma; *Glycyrrhizae* Radix et Rhizoma; *Zingiberis* Rhizoma Recens	Clearing heat and dampness; antioxidant; regulating gut microbiota	Microparticles; particle size: 2775 nm; nanoparticles; particle size: 531 nm	BA; puerarin; BBR; GA	Enhanced absorption; antioxidant effects; protection of pancreatic β-cell function	[123]
Qiyin Sanliang San decoction	*Astragali* Radix; *Lonicerae Japonicae* Flos; *Angelicae Sinensis* Radix; *Glycyrrhizae* Radix et Rhizoma; *Scolopendra*	Clearing heat and toxin; anti-inflammatory	Spherical nanoparticles; Particle size: 240.2 nm; zeta Potential: −7.68 mV	CGA; seco-iridoid glycosides	Suppressed neutrophil infiltration; downregulated CXCL2; promoted tissue repair	[124]
Siwu decoction	*Rehmanniae* Radix Praeparata; *Angelicae sinensis* Radix; Paeoniae Radix Alba; *Chuanxiong* Rhizoma	Nourishing and regulating blood	Spherical or irregular nanoparticles; particle size: 50–500 nm	Proteins; polysaccharides; trace DNA	Promoted hematopoietic repair; protective effect on blood cells; increased blood flow; improved anemia; regulated gene expression	[125]

Huanglian Jiedu Decoction (HJD), a canonical TCM formula comprising *Coptidis* Rhizoma, *Scutellariae* Radix, *Phellodendri chinensis* Cortex, and *Gardeniae* Fructus, has been historically employed for its anti-inflammatory and detoxifying properties, with clinical applications in ischemic brain injury, gastrointestinal disorders, inflammation, and cardiovascular diseases. Chen et al. [94] performed qualitative and quantitative analyses on both supernatant and precipitate phases of HJD, identifying 109 constituent compounds, and quantitative assessment revealed that BA and BBR were markedly enriched in precipitates compared to supernatants. The formation of a self-assembled BA-BBR complex was confirmed, observed as spherical nanoparticles. Zhang et al. [114] subsequently synthesized this complex via precipitation simulation, with pharmacological studies demonstrating its neuroprotection against CoCl₂-induced PC12 cell injury.

BHD, formulated with *Anemarrhenae* Rhizoma, *Glycyrrhizae* Radix et Rhizoma, *Oryzae* Semen, and Gypsum, exerts potent therapeutic effects against fever induced by acute infectious diseases [20]. Lü et al. [20] isolated nanoscale assemblies (N-BHD) enriched with MF, neomangiferin, GA, and ammonium glycyrrhizinate. Notably, MF solubility increased ~ tenfold versus its free form. It was postulated that glycyrrhizin saponins and inorganic ions stabilize N-BHD via micelle formation or electrical double layers, enhancing bioavailability of poorly soluble components like MF. In an LPS-induced febrile rabbit model, N-BHD demonstrated superior antipyretic efficacy and prolonged duration relative to precipitate and dialyzed phases. Further studies demonstrated N-BHD significantly reduced plasma concentrations of pro-inflammatory cytokines, confirming action through inflammatory pathway modulation. Cellular assays confirmed efficient Caco-2 cell uptake, while in vivo studies revealed preferential tissue accumulation in lung and brain. These nanoparticles constitute a critical material basis for BHD's antipyretic efficacy. Ping et al. [115] further established that salinity and conductivity stabilize nanoparticle formation in BHD, identifying *Oryzae* Semen starch as a structural scaffold, with GA enhancing solubility of hydrophobic compounds via reduced surface tension.

Maxing Shigan Decoction (MXSGT), documented in *Shanghan Lun* over two millennia ago, contains *Ephedra* Herba, *Glycyrrhizae* Radix et Rhizoma, *Armeniacae* Semen Amarum, and Gypsum. It is clinically used for cough, fever, pneumonia, and cognate disorders [116]. Zhou et al. [19] isolated 50–150 nm spherical nanoparticles (NPs) from MXSGT, determining that 99.7% of ephedrine and 95.5% of pseudoephedrine were NP-associated rather than freely dispersed. This nanostructure profoundly modulated bioactivity: in normal cells, NPs mitigated ephedrine toxicity, exhibiting cytoprotection. Conversely, in HeLa-229 cancer cells, NPs attenuated ephedrine's pro-proliferative effect. This confirms that self-assembled NPs arising during decocting regulate biological responses by governing active component delivery. Additionally, Pi et al. [21] and Zhao et al. [117] identified metal ions within MXSGT nanoparticles. Removal of any component altered nanoparticle morphology, confirming all herbs participate in assembly. These nanoparticles enriched GA and GRA, exhibiting antipyretic efficacy equivalent to the full decoction via suppression of PGE2, IL-1β, and TNF-α, while modulating serum calcium levels.

Mahuang Fuzi Decoction (MGF), a classical prescription from *Jingui Yaolüe* with documented anti-inflammatory efficacy [118], comprises *Ephedrae* Herba, Glycyrrhizae Radix et Rhizoma, and *Aconiti* Lateralis Radix Praeparata. Yang et al. [119] compared microstructure, composition, and anti-inflammatory activity between co-decocted MGF and a physically admixed combination (MIX) of individually prepared decoctions. Co-decoction induced self-assembly into uniform nanoparticles (MGF SA), whereas MIX formed irregular assemblies. In terms of anti-inflammatory activity, MGF SA showed superior anti-inflammatory effects via NF-κB p65 inhibition and arginine metabolism modulation.

Xiebai San Decoction (XBSD) formulated with *Mori* Cortex, *Lycii* Cortex, *Glycyrrhizae* Radix et Rhizoma, and *Oryzae* Semen, historically applied in Asian countries for pediatric pneumonia. Nie et al. [85] isolated nanoparticles from XBSD (N-XBSD) enriched with mulberroside A/lyciumaside B. N-XBSD enhanced mulberroside A bioavailability and its efficacy is comparable to that of XBSD decoction. Oryzae Semen-derived polysaccharides and proteins likely stabilize nanoparticles, thereby promoting absorption.

Naoluo Xintong Decoction (NLXTD), clinically effective for ischemic stroke [120], consists of six herbs (*Astragali* Radix, *Chuanxiong* Rhizoma, *Notoginseng* Radix et Rhizoma, *Gastrodiae* Rhizoma, *Carthami* Flos, *Angelicae sinensis* Radix) and one animal-derived ingredient (*Scolopendra*). Zhao et al. [121] isolated natural nanoparticles (NLXTD-NPs) comprising polysaccharides, proteins, and saponins. These NLXTD-NPs conferred neuroprotection via suppression of oxidative stress and apoptosis. Critically, removal of these components diminished efficacy, which was restored upon reincorporation, underscoring their biologic indispensability.

Gegen Qinlian Decoction (GQD), a classic TCM formula containing *Puerariae Lobatae* Radix, *Scutellariae* Radix, *Coptidis* Rhizoma, *Glycyrrhizae* Radix et Rhizoma, and *Zingiberis* Rhizoma Recens, utilized for type 2 diabetes and intestinal inflammation [122], yielded micron assemblies and micron-nano assemblies upon isolation [123]. Cellular analysis demonstrated these assemblies markedly enhanced intestinal transport of BA and protected pancreatic β-cells through increased SOD activity, attenuated oxidative damage, and restoration of insulin secretion.

The TCM formula Qiyin Sanliang San Decoction (T-QY305), clinically employed for dermatologic and gastrointestinal disorders, contains *Astragali* Radix, *Lonicerae japonicae* Flos, *Angelicae sinensis* Radix, *Glycyrrhizae* Radix et Rhizoma, and *Scolopendra*. Zhang et al. [124] isolated its nanoparticles (N-QY305). Evaluation in two cancer patients with post-EGFR-inhibitor (EGFRI) dermatitis and in animal models that confirmed T-QY305 significantly alleviated cutaneous inflammation and rash severity. Critically, N-QY305 demonstrated superior therapeutic efficacy relative to T-QY305—achieving enhanced neutrophilic infiltration suppression at equivalent dosing—without compromising EGFRI anticancer activity. These findings establish the decoction-derived assemblies (N-QY305) as fundamental mediators in attenuating EGFRI adverse effects through targeted limitation of CXCL2-guided neutrophil recruitment.

Siwu Decoction (SWD), a traditional blood-regulating formula, finds broad application in addressing anemia, dysmenorrhea, angina, diabetes, and other conditions related to blood deficiency and stasis. It comprises four herbs: *Rehmanniae* Radix Praeparata, *Angelicae sinensis* Radix, Paeoniae Radix Alba, and *Chuanxiong* Rhizoma.Zhang et al. [125] isolated nanoparticles from SWD and demonstrated that these nanoparticles substantially protected the hematological system, promoted hematopoietic function recovery in irradiated zebrafish, and ameliorated phenylhydrazine-induced hemolytic anemia. These findings reveal that nanoparticles present in SWD mediate fundamental effects by modulating key cellular and molecular activities within the hematological system.

In summary, supramolecular assemblies spontaneously formed during the decoction of TCMs (single herbs, herb pairs, or multi-herb formulas) constitute an indispensable material basis for decoction efficacy, achieved through dynamic integration of bioactive components, enhanced bioavailability of key actives, enrichment of synergistic constituents, and optimized pharmacokinetic profiles.

## Supramolecular interpretation of “efficacy enhancement and toxicity reduction” in TCM compatibility theory

The fundamental objective of Traditional Chinese Medicine (TCM) compatibility is to achieve "efficacy enhancement and toxicity reduction" through the strategic combination of herbs [126, 127], while clinical practice has long demonstrated these benefits, modern research seeks to elucidate the material basis underlying these phenomena. Accumulating evidence indicates that supramolecular self-assembly provides a tangible molecular explanation for these therapeutic benefits.

**Fig. 4:**
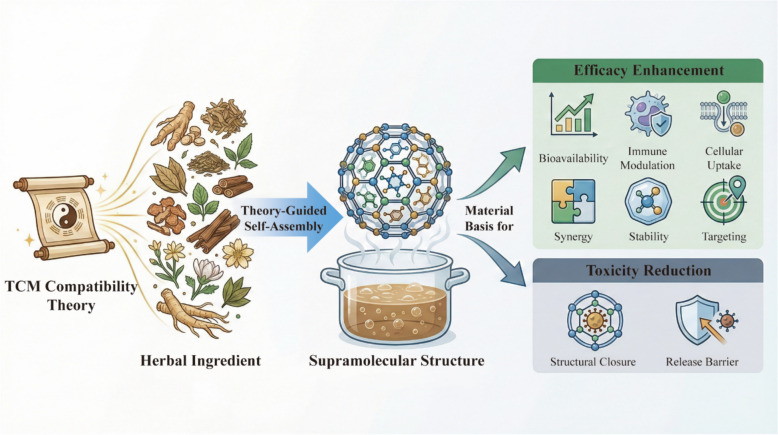
Supramolecular mechanisms underlying “Efficacy Enhancement and Toxicity Reduction” in TCM compatibility

From the perspective of supramolecular chemistry, the interaction between bioactive components in a decoction is not merely a physical mixture but a dynamic process of self-organization driven by non-covalent interactions. This process generates nano/microscale supramolecular assemblies that fundamentally restructure the physicochemical properties of the system. As illustrated in Fig. 4, the mechanisms by which these assemblies translate into "efficacy enhancement and toxicity reduction" operate through well-defined physicochemical and biological pathways. Efficacy Enhancement is primarily achieved through improving solubility, optimizing pharmacokinetics, enhancing cellular uptake, and realizing multi-component synergy. Conversely, Toxicity Reduction is mediated through "structural closure" effects that shield toxic functional groups or by modulating release kinetics to reduce systemic exposure to toxic agents [66, 121]. This focus on supramolecular assembly provides a tangible, evidence-based explanation for how TCM compatibility alters the structural and functional basis of therapeutics at the molecular level.

### Efficacy enhancement

**Fig. 5:**
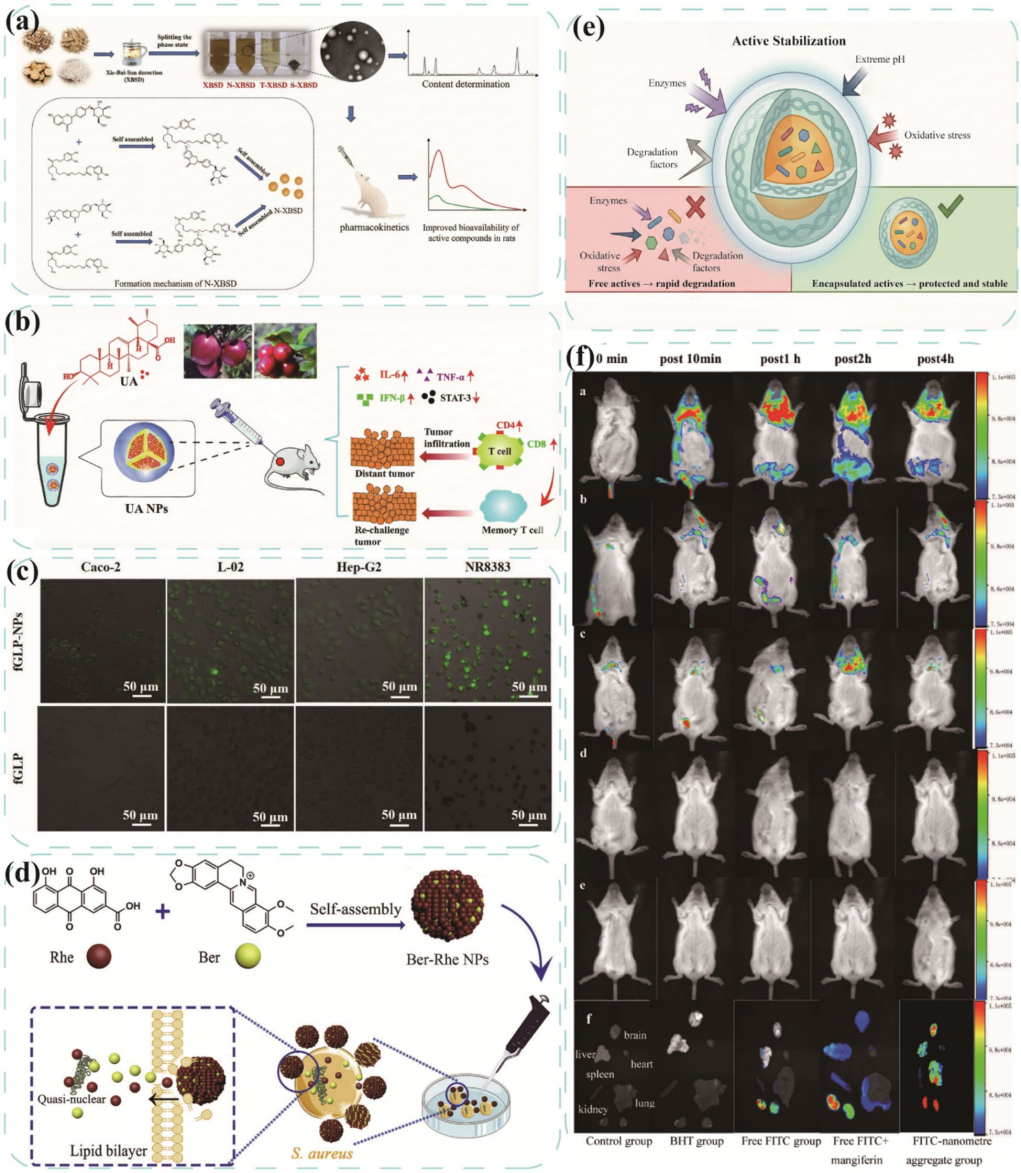
Mechanisms of supramolecular-mediated efficacy enhancement. **a** Bioavailability optimization via Xiebai San nanoparticles (Reproduced from Ref. [85]. Copyright 2024, Dove Medical Press). **b** Immune modulation by Ursolic Acid nanoparticles (Adapted with permission from Ref. [57]. Copyright 2018, American Chemical Society). **c** Enhanced cellular uptake of protein nanoparticles (Reproduced with permission from Ref. [100]. Copyright 2019, American Chemical Society). **d** Synergistic antibacterial effects of Berberine-Rhein assemblies (Reproduced with permission from Ref. [11]. Copyright 2020, Elsevier). **e** Stability improvement of encapsulated components. **f** Tissue-specific targeting to brain and lung (Adapted from Ref. [20]. Copyright 2018, Springer Nature)

Supramolecular assemblies formed through herbal component interactions enhance therapeutic efficacy via the following mechanisms:

1. Bioavailability Enhancement: Supramolecular assemblies optimize bioavailability through dual pathways: (i) Restructuring molecular microenvironments to dramatically improve the solubility of hydrophobic compounds. Illustratively, in BHD assemblies, GA (from *Glycyrrhizae* Radix et Rhizoma) reduces solution surface tension, facilitating dissolution of sparingly soluble MF and neomangiferin (from *Anemarrhenae* Rhizoma). Concurrently, starch macromolecules (from *Oryzae* Semen) form spherical nanoparticles during heating that encapsulate hydrophobic actives, while glycyrrhizin-Ca^2^⁺/Mg^2^⁺ complexes stabilize micellar or bilayered particles, collectively maintaining colloidal dispersion [20, 115]. (ii) Pharmacokinetic profile optimization. Specifically, Nie et al. [85] demonstrated that N-XBSD facilitated mucosal penetration and intestinal absorption, while suppressing P-glycoprotein and multiple efflux transporters to reduce drug efflux. This modulation led to a substantial increase in the elimination half-life of five active ingredients—mulberroside A, kukoamine B, ligiritin, liquiritin apioside, and GA ammonium salt—compared to their free forms (Fig. 5a). Notably, kukoamine B achieved a significantly higher peak plasma concentration than its free counterpart.
2. Immune Modulation and Microenvironment Regulation: Supramolecular assemblies remodel pathological microenvironments through multidimensional biological interactions: (i) Signaling pathway synergy: Self-assembled structures concurrently modulate multiple downstream signal transduction cascades to exert synergistic effects. For example, nanophase assemblies in BHD effectively block inflammatory pathways by inhibiting the expression of key proteins including IL-1β, TRPV4, NF-κB, and TNF-α [115, 128]. Furthermore, UA self-assembled nanoparticles have been demonstrated to block tumor angiogenesis via the COX-2/VEGFR2/VEGFA axis while simultaneously upregulating TNF-α, IL-6, and IFN-β to activate immune responses (Fig. 5b) [57]. (ii) Immune microenvironment remodeling: Functionalized assemblies are capable of reversing immunosuppressive microenvironments and activating immune surveillance. Co-assembled nanoparticles of lentinan (LNT) and UA (LNT-UA NPs) significantly potentiate antitumor immunity by promoting the repolarization of tumor-associated macrophages from the M2 to the M1 phenotype and synergistically blocking the CD47 signal [129]. Similarly, polysaccharide nanoparticles derived from decoctions exhibit immunostimulatory activity by enhancing macrophage phagocytosis and upregulating IL-6 expression [130].
3. Enhanced Cellular Uptake: Nanoscale assemblies leverage size-dependent topological features to facilitate clathrin-mediated endocytosis and membrane fusion, markedly increasing internalization efficiency. For instance, Glycated Licorice Protein (GLP) self-assembled nanoparticles are extensively internalized by the NR8383 macrophage cell line, whereas free GLP lacks this capability (Fig. 5c) [100]. Moreover, nanoparticles mitigate premature clearance resulting from excessively small sizes.
4. Synergistic Component Integration: Dynamic self-assembly enriches functionally complementary actives into optimized therapeutic ensembles. The *Phellodendri Chinensis* Cortex-*Rhei* Radix et Rhizoma combination exemplifies this process: BBR-rhein co-assembled nanoparticles synergistically enhance antibacterial activity (Fig. 5d), reducing the minimum bactericidal concentration against *Staphylococcus aureus* to 0.1 μmol/mL and improving biofilm eradication by 2.5-fold [11].
5. Active Stabilization: Supramolecular encapsulation provides physical barriers against enzymatic degradation, pH extremes, and oxidative stress (Fig. 5e). When *Anemarrhenae* Rhizoma and *Phellodendri Chinensis* Cortex combine, MF-BBR-polysaccharide nanoparticles maintain structural integrity for > 12 h in simulated gastric and intestinal fluids, whereas nonencapsulated controls degrade within 4 h [110]. Analogously, HJD nanoparticles sequester actives within hydrophobic cores, limiting environmental exposure [94].
6. Targeted Delivery: Size- and surface-tuned assemblies enable tissue-specific biodistribution. BHD nanoparticles preferentially accumulate in brain and lung tissues (Fig. 5f) [20, 115], thereby amplifying therapeutic indices while reducing off-target effects.

Collectively, supramolecular reorganization systemically potentiates decoction efficacy. By integrating mechanisms such as bioavailability optimization, immune microenvironment regulation, and targeted delivery with synergistic component assembly and active stabilization, these structures establish a mechanistic foundation for traditional TCM formulation principles.

### Toxicity reduction

**Fig. 6:**
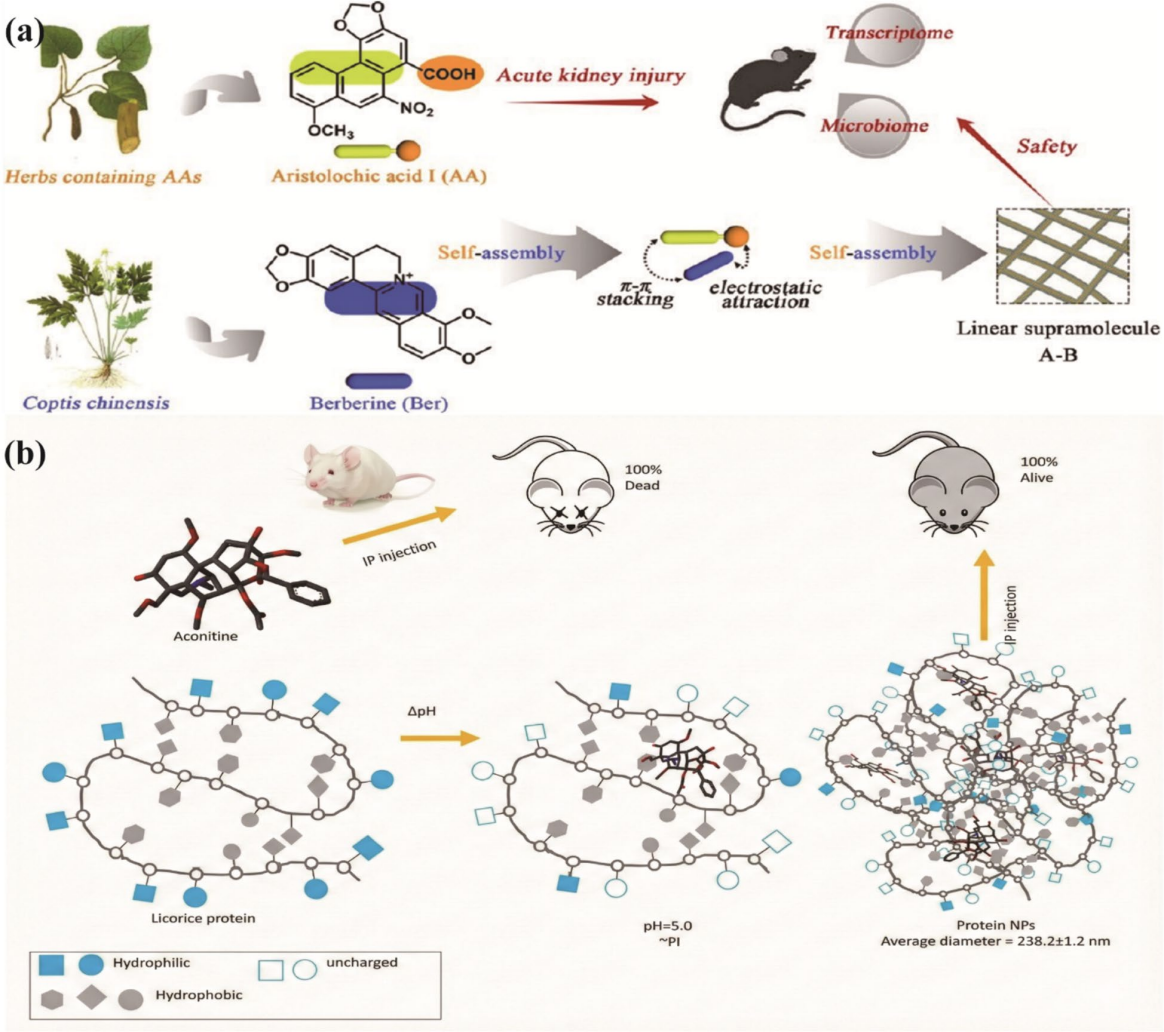
Strategies for toxicity reduction via supramolecular assembly. **a** "Structural Closure" masking toxic groups in Aristolochic Acid (Reproduced with permission from Ref. [66]. Copyright 2021, American Chemical Society). **b** Bioavailability reduction via encapsulation of Aconitine in protein nanoparticles (Reproduced from Ref.[13]. Copyright 2015, Springer)

The inherent complexity of TCM chemical profiles means certain active components harbor toxic side effects alongside their therapeutic benefits. Classic TCM pairings leverage long-established toxicity-reduction mechanisms, exemplified by practices such as “toxicity restraint” and “detoxification.” For instance, pairing *Notoginseng* Radix et Rhizoma with *Glycyrrhizae* Radix et Rhizoma mitigates hepatotoxicity; *Pinelliae* Rhizoma coupled with *Zingiberis* Rhizoma Recens substantially diminishes throat mucosal irritation; furthermore, highly toxic *Aconiti* Lateralis Radix Praeparata achieves enhanced safety through combination with *Zingiberis* Rhizoma and *Glycyrrhizae* Radix et Rhizoma. Accumulating evidence demonstrates that supramolecular structures formed by TCM components potently reduce toxicity. Primary mechanistic pathways include:

1. “Structural Closure” of Toxic Functional Groups: Self-assembled supramolecular structures encapsulate, shield, or modify exposed conformations of deleterious moieties within toxic molecules. This “structural closure” effectively lowers the probability of direct interaction between toxic groups and biological targets and attenuates chemical reactivity by reconfiguring intermolecular interaction networks. Aristolochic acid (AA), associated with pathologies including acute kidney injury, aristolochic acid nephropathy, and hepatic carcinoma [131], exemplifies this principle. Specifically, TCM formulations containing AA paired with BBR-rich herbs exhibit markedly reduced toxicity, with the combination of AA-containing herbs and *Coptidis* Rhizoma (BBR source) ensuring clinical safety. Corroborating this, Wang et al. [66] demonstrated in zebrafish and murine models that BBR-AA hetero-supramolecular self-assembly significantly mitigates AA toxicity and ameliorates AA-induced acute kidney injury. Mechanistic analysis revealed linear hetero-supramolecules (A-B) formed via electrostatic attraction and *π-π* stacking, displaying external hydrophobic groups and internal hydrophilic groups. This structural arrangement obstructs AA’s toxic sites and inhibits its metabolic activation. Moreover, unlike free AA, the A-B supramolecule preserves gut microbiota homeostasis. Consistent RNA sequencing immunoblots and immunofluorescence analyses of murine renal tissue confirmed that the supramolecular complex virtually abolishes AA’s activation of immune and carcinogenic pathways (Fig. 6a).
2. Reduced Bioavailability of Toxic Molecules: Supramolecular assemblies generate physical barriers encapsulating toxicants, diminishing or delaying their release and absorption kinetics. This curtails systemic bioavailability and associated toxicity. *Glycyrrhizae* Radix et Rhizoma -*Aconiti Kusnezoffii* Radix pairing illustrates this mechanism: glycyrrhiza protein (GP) self-assembles into nanoparticles under acidic conditions, entrapping Aconitum alkaloids (AC). Ke et al. [13] conducted murine acute toxicity studies, observing that free AC induces severe toxicity leading to rapid lethality, whereas AC encapsulated in GP nanoparticles (GP-AC NPs) elicits only mild transient symptoms followed by full recovery within hours (Fig. 6b). Collectively, these findings confirm that GP nanoparticles significantly impede AC bioavailability and toxicity by delaying molecular interactions between toxicants and biological targets.

## Biomedical applications of supramolecular self‑assembly in TCM

**Fig. 7:**
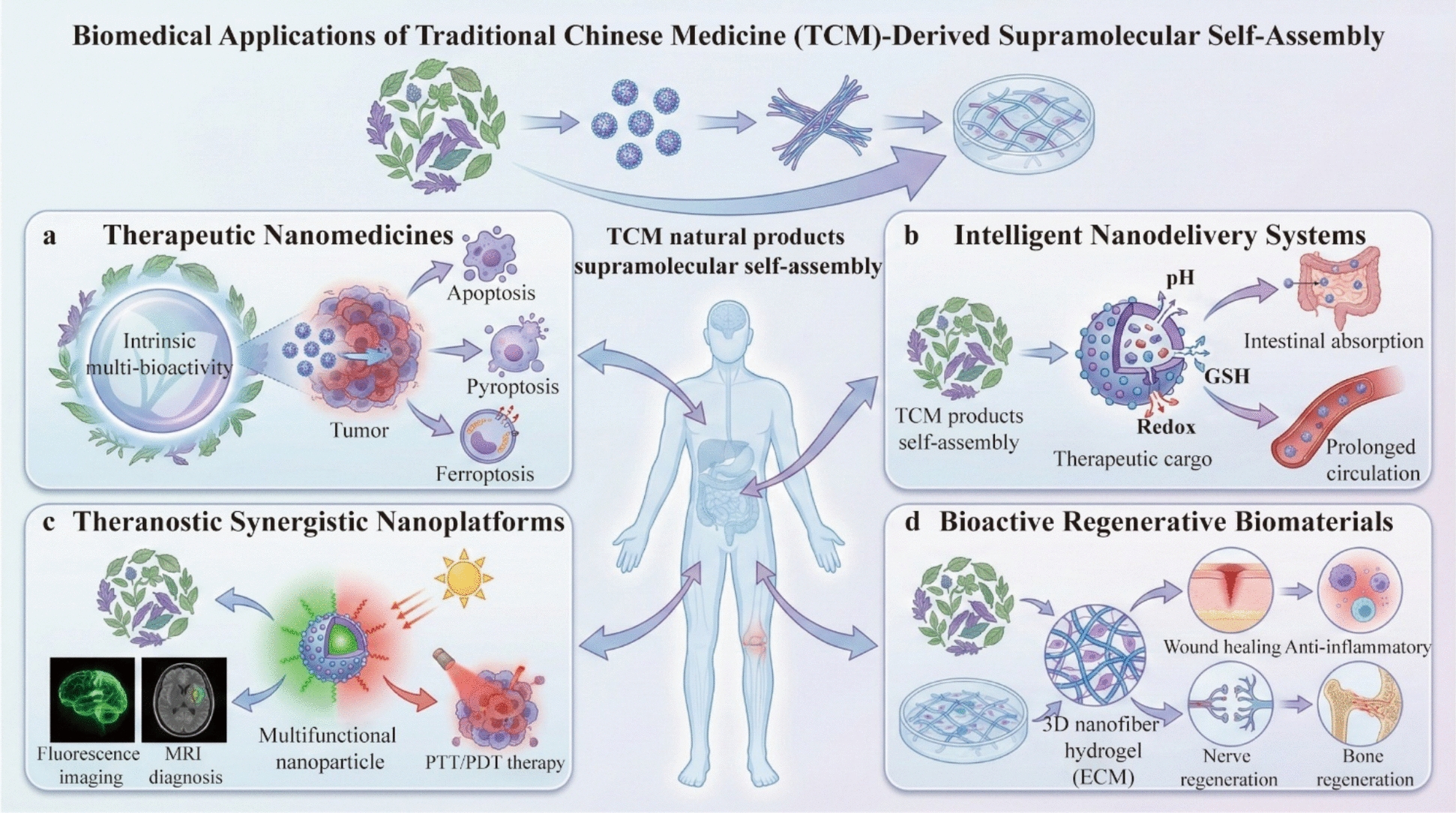
Schematic representation of key biomedical applications of TCM supramolecular self-assembly technology: **a** Therapeutic nanomedicines; **b** Intelligent nanodelivery systems; **c** Theranostic synergistic nanoplatforms; **d** Bioactive regenerative biomaterials

Bridging ancient herbal wisdom with modern nanotechnology, TCM-derived supramolecular self-assembly technology is driving transformative progress in biomedicine. Within the complex matrix of herbal systems, diverse bioactive components—harnessing intrinsic molecular recognition and self-organization capabilities—assemble into hierarchically ordered nanostructures via non-covalent interactions, establishing the molecular foundation for biomedical innovation. Unlike synthetic carriers employed in conventional drug development, phytochemical-based supramolecular systems offer unique biomimetic properties: transcending bioavailability limitations of traditional formulations, conferring stimulus-responsive behavior, and enabling synergistic therapeutic efficacy—thereby pioneering novel approaches for precision medicine. Critically, these assemblies serve dual roles as both delivery vectors and active therapeutics, epitomizing the “carrier-as-drug” paradigm. Their multifunctionality spans targeted delivery, disease imaging, and regenerative medicine (Fig. 7). This section analyzes four cutting-edge biomedical applications, elucidating how TCM components function not merely as payloads, but as functional supramolecular building blocks to overcome barriers in pharmaceutical R&D.

### Therapeutic nanomedicines

Supramolecular self-assembly enables the development of next-generation therapeutics by precisely leveraging the synergistic nature of TCM components. This approach mirrors the TCM principle of "compatibility" (*Jun-Chen-Zuo-Shi*) at the nanoscale, where monomers are co-assembled to achieve multidimensional empowerment: (1) Drug Delivery Enhancement: Self-assembled herbal nano-clusters integrate multifunctional capabilities—improving the bioavailability of difficult-to-deliver natural products. (2) Spatiotemporal Therapeutic Synergy: Dynamically dissociable components enable site-specific release, achieving synergistic effects unattainable with single-molecule synthetic drugs.

These self-assembled therapeutics exhibit substantially enhanced bioavailability. For instance, reflecting the synergy of TCM combinations, Jiang et al. [132] developed reduction-sensitive dual-drug nanoparticles from a hydrophobic conjugate of TCM-derived paclitaxel and Vitamin E succinate with tetrandrine (a bisbenzylisoquinoline alkaloid from *Stephania tetrandra*). Upon cellular internalization, the disulfide bond cleavage releases the drugs, while tetrandrine—acting beyond a mere structural component—potentiates therapy through ROS induction and P-glycoprotein inhibition. This system achieved synergistic therapy against multidrug-resistant breast cancer. Huang et al. [62] fabricated a "natural self-assembled herbal pair" using BBR (from *Coptis chinensis*) and CA (from *Cinnamomum cassia*). The CA-BBR NPs demonstrated potent antibacterial activity and biofilm eradication. By harnessing the intrinsic antimicrobial properties of these TCM alkaloids and organic acids, the system offers a novel strategy against drug-resistant bacteria. Lin et al. [133] created a coordination-crosslinked nanoassembly (FPSN, Fe^3^⁺-Piceatannol-Sorafenib Nanoassembly) using Piceatannol (a natural stilbenoid analogous to resveratrol), Fe^3^⁺, and Sorafenib. Leveraging the polyphenol-metal coordination common in herbal processing, Fe^3^⁺-Piceatannol (PCT) coordination drives self-assembly. FPSN integrates photothermal therapy with ferroptosis induction, overcoming resistance in hepatocellular carcinoma. Luo et al. [134] revealed that Oleanolic Acid (OA), a ubiquitous pentacyclic triterpenoid in TCM, undergoes critical concentration-triggered self-assembly into stable nanomicelles. Exploiting OA's intrinsic bioactivity, these micelles directly target the PSMA6 proteasome subunit to elicit tumor cell pyroptosis. Li et al. [135] further demonstrated that ethanol-mediated self-assembly of this TCM triterpenoid generates nanostructures with superior tumor penetration. This pure TCM-component system significantly enhanced antitumor efficacy by remodulating the tumor microenvironment, advancing the precision design of carrier-free herbal nanodrugs.

### Intelligent nanodelivery systems

Conventional small-molecule drugs and synthetic polymer carriers often face translational barriers including toxicity and poor biodegradability [136, 137]. In contrast, supramolecular delivery systems fabricated directly from natural phytochemicals offer a "green" alternative with inherent biocompatibility. These TCM-based nanoplatforms establish a functional “delivery-synergistic therapy” paradigm, where the carrier itself contributes to the therapeutic outcome.

For instance, utilizing the metal-coordination ability of Cur (the polyphenol from *Curcuma longa*), nanoparticles were fabricated by integrating 9-fluorenylmethoxycarbonyl-L-histidine, Zn^2^⁺, and Cur [71]. Cur acts not only as the payload but also participates in structural stabilization, achieving high drug loading and pH/GSH-responsive release with potent antitumor efficacy. Similarly, Chen et al. [26] fabricated core–shell nanoparticles through hetero-assembly of two renowned TCM components: (-)-epigallocatechin-3-gallate (EGCG, from green tea) and a hydrophobic Cur core, stabilized by Polyvinylpyrrolidone (PVP). By exploiting the intermolecular hydrogen bonding typical of polyphenols, CEP-NPs achieve intestine-targeted release and a 12-fold increase in oral bioavailability. Dai, L. et al. [138] utilized Ginsenoside Rb1 (the major saponin of *Panax ginseng*) as a natural supramolecular scaffold to co-assemble betulinic acid and other agents. Capitalizing on the amphiphilic structure of Ginsenoside Rb1, these assemblies enhance drug solubility and prolong systemic persistence. Rb1’s intrinsic bioactivity further affords synergistic benefits, exemplifying a simplified, eco-compatible fabrication strategy using TCM saponins.

### Theranostic synergistic nanoplatforms

The inherent modularity of supramolecular self-assembly offers a natural molecular scaffold for multimodal theranostics. Many TCM components possess intrinsic optical properties or unique chemical structures that facilitate the integration of diagnostic and therapeutic functions.

For instance, Zhang et al. [139] fabricated core–shell nanoparticles through the co-assembly of a derivative of Hypocrellin (a perylenequinone pigment from the TCM fungus *Hypocrella bambusae*) with human serum albumin. Leveraging the natural photosensitivity of the Hypocrellin structure, these NPs exhibit high photothermal conversion efficiency, enabling dual-modal imaging and synergistic therapy. Deng et al. [140] similarly designed a Hypocrellin B-based system, modifying this natural fungal pigment to create liposome-photosensitizer complexes with enhanced solubility for microvascular disease therapy. Zhao et al. [141] engineered a carrier-free nanodrug via the green co-assembly of UA (a triterpenoid widely distributed in herbs like *Prunella vulgaris*), lactobionic acid, and Indocyanine Green. UA serves as the structural foundation, generating nanoparticles that boost intracellular accumulation and provide an integrated cancer theranostic approach. An et al. [142] synthesized Dehydroberberine (a derivative of the TCM alkaloid BBR) and co-assembled it with tetraphenylborate. By exploiting the planar conjugate structure of the BBR skeleton, the system delivers the drug to mitochondria and emits intense Near-infrared (NIR) fluorescence, achieving tumor suppression while monitoring mitochondrial dynamics. Ge et al. [143] exploited the specific binding of boronic acid to β-D-glucan (a polysaccharide common in medicinal mushrooms) to synthesize core–shell nanoparticles. This system preserves the immunomodulatory activity native to the glucan, providing dual-modal imaging while establishing a paradigm for polysaccharide-based multifunctional theranostics.

### Bioactive regenerative biomaterials

Distinct from the discrete nanostructures of particulate delivery systems discussed above, supramolecular hydrogels represent a unique class of macroscopic soft materials formed through the hierarchical self-assembly of TCM components into three-dimensional fibrous networks. Due to their high water content and porous architecture, these TCM-based hydrogels structurally mimic the natural extracellular matrix (ECM), making them ideal candidates for tissue engineering and regenerative medicine. Unlike synthetic hydrogels that often require the addition of exogenous growth factors, TCM supramolecular hydrogels act as "bioactive scaffolds," where the building blocks themselves exert pharmacological effects—such as anti-inflammatory or antimicrobial activities—to accelerate tissue repair and regeneration.

Li et al. [144] developed a hybrid hydrogel based on GA, the primary bioactive saponin of Licorice (*Glycyrrhiza glabra*). The self-assembled core utilizes GA’s fibrillar network forming ability. Crucially, the hydrogel retains GA’s inherent anti-inflammatory and antimicrobial activities, functioning as an advanced wound dressing that accelerates healing. Zou et al. [145] employed the self-assembly of Glycyrrhetinic Acid (GRA, the aglycone of GA) to fabricate a sprayable hydrogel. Harnessing GRA’s natural anti-inflammatory role, the gel attenuates pro-inflammatory mediators (TNF-α, PGE₂) and prevents postoperative adhesions. Zheng et al. [9] reported a directed self-assembly hydrogel based on rhein, an anthraquinone from Rhubarb. This rhein hydrogel forms a nanofibrous network that provides a biomimetic scaffold for neural tissue. By sustainably releasing rhein and effectively inhibiting neuroinflammation via the TLR4/NF-κB pathway, the hydrogel not only protects neurons but also creates a conducive microenvironment for neural regeneration.

## Conclusion

The complexity of the material basis of efficacy in TCM extends far beyond single chemical components, with multi-component synergistic effects and the resulting supramolecular structures playing critical roles. This review systematically synthesizes the latest advances in TCM supramolecule research, aiming to provide novel perspectives for deeply elucidating the material basis of TCM efficacy and the scientific implications of compound formulations.

Initially, grounded in supramolecular chemistry principles, this work expounds on the molecular mechanisms driving self-assembly within complex TCM systems. It dissects the cooperative non-covalent interactions—such as hydrogen bonding, electrostatic interactions, and *π-π* stacking—that dictate assembly behaviors. A comprehensive summary establishes the intrinsic connection between these forces and the structural characteristics of key TCM components, ranging from small molecules (quinones, flavonoids, terpenoids, saponins, alkaloids, polyphenols) to metal ions, biomacromolecules (proteins, polysaccharides, nucleic acids), and the notably emerging ELNVs. To enable the study of these complex systems, the review also outlines the essential separation strategies and multimodal characterization techniques that provide the methodological foundation for identifying and validating these supramolecular architectures.

Subsequently, the review transitions from the molecular level to the macroscopic decoction system, elucidating the physical morphology and pharmacological roles of supramolecular assemblies across the hierarchy of single herbs, herb pairs, and multi-herb formulas. Evidence demonstrates that these assemblies are not merely byproducts but constitute a bioactive material basis directly correlated with the therapeutic efficacy of the corresponding decoctions. Crucially, this review elucidates the role of supramolecular assembly as the structural basis for the classic TCM objective of "efficacy enhancement and toxicity reduction." The formation of supramolecular assemblies serves as a dynamic "composition-structure–function" coupling mechanism. Through strategies such as "structural closure" of toxic groups, solubility enhancement, and multi-component synergy, these assemblies fundamentally modulate the bioavailability, stability, targeting capability, and toxicity profiles of active ingredients, thereby materializing the traditional wisdom of compatibility at the molecular level. Ultimately, bridging ancient wisdom with modern innovation, the review highlights the vast translational potential of TCM component self-assembly in biomedicine. Leveraging the "carrier-as-drug" paradigm and the inherent biocompatibility of natural products, TCM supramolecular technology has successfully driven the development of therapeutic nanomedicines, intelligent stimulus-responsive delivery systems, multifunctional theranostic platforms, and bioactive regenerative biomaterials. These advancements not only validate the scientific substance of TCM but also pioneer new pathways for the development of precision natural medicines.

Despite significant progress in TCM supramolecule research, crucial challenges remain, alongside substantial opportunities:

1. Need for High-Resolution Dynamic Characterization and Elucidation of Internal Fate: Current research is predominantly confined to static in vitro physicochemical characterization, primarily focusing on the observation of surface morphology and particle size. However, a critical gap exists in understanding the dynamic fate and deep molecular mechanisms of supramolecular assemblies after entering the body. Specifically, the real-time changes in in vivo assembly/disassembly kinetics, mechanisms of crossing biological barriers, intracellular localization and metabolic pathways, and complex interactions with endogenous biological macromolecules remain largely a "black box." Furthermore, the molecular mechanisms governing long-term in vivo safety are not fully elucidated. Developing novel high-resolution, label-free in situ imaging techniques is urgently needed to shift focus from surface characteristics to revealing these authentic, dynamic supramolecular processes within physiological environments.
2. Evolution from Binary Models to Multi-Component Complexity: Currently, the majority of TCM supramolecular research relies on simplified binary models involving the co-assembly of two specific compounds. While this represents a pivotal advancement over reductionist single-component studies, it barely scratches the surface of the immense complexity inherent in TCM decoctions, which contain hundreds of coexisting constituents. The real-world decoction environment involves simultaneous, competitive, and cooperative interactions among multiple components. Relying solely on dual-component interaction studies is insufficient to fully capture the holistic self-assembly landscape of actual decoctions. Future research must transcend these idealized systems to investigate complex multi-component supramolecular networks, elucidating how specific "bystander" components or the decoction matrix regulate the assembly process, thereby approaching the true material basis of TCM.
3. Integration of Computational Tools and AI: The self-assembly of TCM supramolecular systems is governed by the concerted interplay of diverse phytochemical structures, environmental conditions, concentration, and coexisting components. Currently, universal quantitative structure–property relationship models and efficient predictive tools are lacking for correlating the structural features of complex TCM components (especially multi-component decoctions) with the ultimate properties of their assemblies. Future efforts should deeply integrate multiscale computational strategies, ranging from static quantum mechanical (QM) calculations for rationalizing intermolecular non-covalent interactions to MD simulations (utilizing both top-down and bottom-up approaches) for visualizing the dynamic pathways of TCM self-assembly [146–148]. Furthermore, the application of artificial intelligence (AI) and machine learning algorithms will be pivotal in analyzing vast datasets to establish quantitative structure–property relationships (QSPR) [149, 150]. These computational tools can accelerate the discovery of novel bioactive supramolecular structures and predict assembly propensity, thereby guiding the rational design of modernized, function-oriented TCM formulations [151, 152].
4. Potential Side Effects and Nanotoxicity: Although supramolecular assemblies derived from traditional Chinese medicine components are generally considered biocompatible and of low toxicity due to their natural origin, their nanoscale properties—such as altered biodistribution, prolonged circulation time, and enhanced cellular uptake—could lead to unintended side effects. For instance, nano-bio interface interactions may induce oxidative stress, inflammatory responses, or unintended accumulation in non-target organs, raising concerns about long-term safety and nanotoxicity. Furthermore, the dynamic and reversible nature of supramolecular structures may lead to unpredictable dissociation and reorganization in vivo, potentially exposing toxic functional groups or altering metabolic pathways. Therefore, systematic toxicological profiling and long-term biosafety studies must be conducted prior to clinical translation.
5. Development of Intelligent, Multifunctional Supramolecular Materials: Capitalizing on the biological activity and self-assembly properties of TCM components, efforts should focus on designing and developing reversible self-assembled/self-disassembled systems responsive to environmental stimuli (e.g., pH, enzymes, redox, light/heat), such as smart hydrogels and nanocarriers. This will broaden their translational applications in targeted tumor therapy, precision drug delivery, and tissue repair and regenerative medicine.
6. Regulatory and Scalability Challenges for Clinical Translation: The transition of TCM supramolecular systems from bench to bedside faces rigorous regulatory and manufacturing hurdles. The inherent heterogeneity of botanical raw materials (due to seasonal or geographical variations) leads to significant challenges in Chemistry, Manufacturing, and Controls (CMC), particularly regarding batch-to-batch inconsistency in nanoparticle size, charge, and drug loading, which complicates quality control and reproducibility [153, 154]. To mitigate these variations, recent studies have proposed a "controlled assembly" strategy—such as decoupling the extraction process from the self-assembly phase via pH regulation. This approach has proven effective in constructing highly uniform and stable supramolecular nanoparticles, thereby overcoming the limitations of spontaneous "extraction-assembly integration" and ensuring predictable therapeutic efficacy [155]. From a regulatory perspective, current frameworks often lack harmonized guidelines tailored for complex botanical nano-formulations. This creates hurdles in defining critical quality attributes (CQAs) and demonstrating bioequivalence, thereby delaying approval pathways [156, 157]. Successful clinical translation requires establishing robust, scalable manufacturing processes adhering to Good Manufacturing Practice (GMP) standards, alongside advanced physicochemical characterization to ensure the structural integrity and therapeutic reproducibility of the final product.
7. Deepening Integration with TCM Theory: While some scholars have proposed supramolecular interpretations of fundamental TCM theories, such as medicinal property theory, meridian theory, and formulation theory, based on related studies [18], sustained exploration is still required. Critical areas include establishing methods for studying in vivo supramolecular chemistry, clarifying the supramolecular essence of meridians and visceral systems, developing supramolecular characterization systems linking TCM's microscopic matter to macroscopic states, and constructing predictive models of compound compatibility patterns. The ultimate goal is to integrate these insights into a modern theoretical framework rooted in supramolecular chemistry.

With continued breakthroughs in advanced characterization techniques, multiscale computational simulations, and intelligent material design, supramolecular chemistry is poised to inject powerful momentum into elucidating the scientific basis of TCM theories, accelerating the modernization of TCM, and fostering the development of innovative therapeutics and advanced biomaterials derived from natural products, ushering in a new era for natural medicine research.

## Data Availability

No datasets were generated or analysed during the current study.

## Abbreviations

AA: Aristolochic acid
AC: Aconitum alkaloids
AI: Artificial intelligence
APS: *Angelicae sinensis* Radix polysaccharides
BA: Baicalin
BBR: Berberine
BHD: Baihu decoction
BLGD: Isatidis Radix (Ban-Lan-Gen) decoction
CA: Cinnamic acid
CEP-NPs: Cur-EGCG-PVP nanoparticles
CGA: Chlorogenic acid
CMC: Chemistry, manufacturing, and controls
CQAs: Critical quality attributes
cryo-EM: Cryo-electron microscopy
Cur: Curcumin
DLS: Dynamic light scattering
ECM: Extracellular matrix
EGCG: Epigallocatechin-3-gallate
EGFRI: EGFR-inhibitor
ELNVs: Exosome-like nanovesicles
EndMT: Endothelial-to-mesenchymal transition
EPR: Enhanced permeability and retention
FPSN: Fe^3^⁺-Piceatannol-Sorafenib nanoassembly
FTIR: Fourier-transform Infrared
Full-API: Full-active pharmaceutical ingredient
GA: Glycyrrhizic acid
GLP: Glycated licorice protein
GMP: Good manufacturing practice
GP: Glycyrrhiza protein
GQD: Ge-Gen-Qin-Lian decoction
GRA: Glycyrrhetinic acid
GSH: Glutathione
HJD: Huanglian Jiedu decoction
HK: Honokiol
HST: Hesperetin
IL-1β/IL-6/TNF-α: Interleukin-1 Beta/Interleukin-6/Tumor Necrosis Factor-Alpha
ITC: Isothermal titration calorimetry
LNT: Lentinan
LNT-UA NPs: Lentinan-Ursolic acid nanoparticles
MD: Molecular dynamics
MF: Mangiferin
MGF: Mahuang Fuzi decoction
MXSGT: Maxing Shigan decoction
N-BHD: Nanoscale assemblies of Baihu decoction
N-QY305: Nanoparticles of Qiyin Sanliang San decoction
N-XBSD: Nanoparticles of Xiebai San decoction
NIR: Near-infrared
NLXTD: Naoluo Xintong decoction
NLXTD-NPs: Nanoparticles of Naoluo Xintong decoction
NMR: Nuclear magnetic resonance
NPs: Nanoparticles
OA: Oleanolic acid
PCT: Piceatannol
PDEVs: Plant-derived extracellular vesicles
PGE2: Prostaglandin E2
P-gp: P-glycoprotein
PVP: Poly(N-vinylpyrrolidone)
QM: Quantum mechanical
QSPR: Quantitative structure–property relationships
ROS: Reactive oxygen species
SA: Supramolecular self-assembly
SAN: Sanguinarine
SEC: Size exclusion chromatography
SEM: Scanning electron microscopy
SH: *Scutellariae* Radix-*Hedyotidis* Herba pair
SH-NPs: Nanoparticles of *Scutellariae* Radix-*Hedyotidis* Herba pair
SOD: Superoxide dismutase
SWD: Siwu decoction
T-QY305: Qiyin Sanliang San decoction
TAMs: Tumor-associated macrophages
TCM: Traditional Chinese Medicine
TEM: Transmission electron microscopy
TME: Tumor microenvironment
UA: Ursolic acid
UV: Ultraviolet
XBSD: Xiebai San decoction
YQHXJDD: Yiqi Huoxue Jiedu decoction
